# Analysis of gas bubbling dynamics via lie symmetry approach of nonlinear wave equation

**DOI:** 10.1038/s41598-025-09588-6

**Published:** 2025-08-09

**Authors:** Ehab M. Almetwally, Rasha Saleh, Samah M. Mabrouk, Ahmed S. Rashed

**Affiliations:** 1 Department of Mathematics and Statistics, College of Science, Imam Mohammad Ibn Saud Islamic University (IMSIU), 11432 Riyadh, Saudi Arabia; 2 Department of Physics and Engineering Mathematics, Faculty of Engineering, Zagazig University, Zagazig, Egypt

**Keywords:** Nonlinear wave equation, Lie Infinitesimals, Optimal system, Solitons, Mathematics and computing, Physics

## Abstract

Bubbles formed by the introduction of gas into a liquid are a common phenomenon, known as gas bubbling in liquids. This process is widely utilized in various industries for aeration, mixing, and purification. An optimal system of Lie symmetry analysis is employed to investigate the generalized (3 + 1)-dimensional nonlinear wave equation (NLWE). Single, double, triple, and quadruple linear combinations are constructed to derive novel solutions that represent different dynamic and turbulent behaviors of the bubbles. This equation models a wide range of nonlinear phenomena occurring in liquids containing gas bubbles. The proposed methodology is used to obtain a diverse set of accurate soliton solutions to the equation. Furthermore, the resulting solutions are analyzed in terms of their physical interpretations.

## Introduction

Gas bubbling in liquids is a common phenomenon that occurs when a gas is introduced into a liquid, resulting in the formation of bubbles. This process is widely employed across various industries for mixing, aeration, and purification purposes. The characteristics of the formed bubbles, such as their size and frequency, depend on several factors, including gas flow rate, liquid viscosity, and surface tension. Overall, gas bubbling in liquids plays a crucial role in many industrial processes, rendering it a subject of significant interest to researchers and engineers alike^1^. Understanding the nonlinear and shock wave dynamics involved in gas bubbling is essential for optimizing these industrial applications. Detecting and analyzing these phenomena can enhance the performance of mixing and aeration systems and improve the quality control of purified liquids. By gaining a deeper understanding of gas–liquid interactions under various conditions, researchers can devise more effective strategies for controlling and optimizing these processes. In essence, the investigation of nonlinear and shock wave dynamics in gas bubbling is fundamental to the advancement of industrial fluid dynamics.

Bubbles, regarded as inhomogeneities^2^, play a critical role in influencing the nonlinear properties of the medium. Consequently, significant efforts have been made from various perspectives to measure the distribution of bubble sizes^3^. These efforts involve the application of acoustic and optical techniques, often combined with extensive computational analysis. Inhomogeneities are also present in the ocean, occurring on a much larger scale than individual bubbles. Although they may not be as visually apparent, their impact on sound propagation is considerable due to the vast distances over which sound travels in the marine environment. Local variations in sound speed arise from the mixing of water masses with different temperatures and salinities. Additionally, oceanic features such as currents, tides, eddies, and internal waves further contribute to the presence of these inhomogeneities.

The (3 + 1)-dimensional nonlinear wave equation (NLWE) can be defined as^4^:

1 $${\left({u}_{t}+{a}_{2} {u}_{xxx}+{a}_{1}u{u}_{x}+{a}_{3}{u}_{x}\right)}_{x}+{a}_{4}{u}_{yy}+{a}_{5}{u}_{zz}=0.$$

This equation provides a framework for modeling the complex interactions between waves and gas bubbles in a liquid. By incorporating the nonlinear advection term, $u{u}_{x}$, the dispersion term, ${u}_{xxx}$, and multidimensional effects, the equation can capture a wide range of nonlinear physical processes, such as wave–bubble interactions, bubble oscillations, sonoluminescence, and cavitation. As such, it serves as a powerful tool for investigating the rich and complex dynamics of bubbly liquids across various scientific and engineering applications^5,6^. The nonlinear wave equation (NLWE) described in Eq. (1) has been previously examined using various analytical techniques. Hirota’s bilinear method^5^ was applied to determine lump soliton and its interaction solutions for Eq. (1) revealing two distinct dynamical behaviors: general lump–periodic and multi-kink soliton solutions. The stability of this model is examined by using the modulation instability analysis^6^, then the dynamical behavior and solutions are discussed through generalized exponential rational function method. Furthermore, Lie symmetry, multiplier, and simplest equation methods were employed on Eq. (1), leading to novel symmetry reductions, group-invariant solutions, and conservation laws^7^. Multi-soliton and periodic solutions for the NLWE with variable coefficients were reported in^8^. In addition, the linear superposition principle was used to explore special N-wave, resonant multiple wave, and complexion solutions for the generalized (3 + 1)-dimensional nonlinear wave equation describing gas bubbling in liquids, as presented in^9^. Generally, nonlinear wave equations in multiple dimensions have been extensively studied using a variety of analytical techniques. Sine–Cosine^10–14^, tanh-coth^12,15–19^, inverse scattering^20–22^, Hirota bilinear^23–28^, extended homogenous method^29–35^, Exp-function^36–39^, Elliptic function^40–46^, Bäcklund transformation^47^, Darbaux Transformation^48,49^ symmetry transformations and singular manifolds methods^50–61^ have also been employed to investigate the behavior of nonlinear partial differential equations (PDEs).

Previous research on gas bubbling dynamics has primarily concentrated on the behavior of bubbles in stagnant liquids, with relatively limited attention given to the effects of gas bubbling on fluid flow and mixing. This research paper builds upon existing knowledge by investigating how gas bubbling influences the flow patterns and turbulence levels within a liquid medium. A more comprehensive understanding of these dynamics can enable engineers to optimize process parameters, thereby improving mixing and aeration efficiency in industrial applications. The present research aims to explore the complex dynamics of gas bubbling in liquids and their implications for industrial processes. By examining the nonlinear and shock wave phenomena that arise during bubble–liquid interactions, engineers can develop more effective and energy-efficient systems for mixing and aeration. Ultimately, the goal is to enhance the overall performance, reliability, and scalability of industrial processes that rely on gas–liquid interactions. To this end, the generalized (3 + 1)-dimensional nonlinear wave equation (NLWE) is analyzed for exact solutions, including single and multiple solitons, periodic, logarithmic, exponential, and polynomial wave forms. Additionally, certain solutions are expressed in terms of arbitrary functions, which can be appropriately selected to model various physical scenarios. While the NLWE has been previously studied using different analytical techniques, the current approach, based on the Lie optimal system, yields new exact solutions that have not been reported in earlier works.

The structure of the paper is as follows. In Section “Lie transformations of NLWE”, the Lie symmetry vectors of the (3 + 1)-dimensional nonlinear wave equation (NLWE) are presented. Section “Optimal system of lie vectors” is dedicated to the reduction of the NLWE using the optimal Lie symmetry vectors. Finally, the paper concludes with a summary of the main findings in Section “Discussion of the results”.

## Lie transformations of NLWE

The (3 + 1)-dimensional NLWE has the following Lie infinitesimals:

2 $$\begin{aligned} X_{1} = & \,\left( {\frac{{ - 1}}{{2a_{4} }}y\frac{d}{{dt}}f_{1} \left( t \right) - \frac{1}{{2a_{5} }}z\frac{d}{{dt}}f_{2} \left( t \right) + f_{3} \left( t \right)} \right)\frac{\partial }{{\partial x}} \\ & \quad + f_{1} \left( t \right)\frac{\partial }{{\partial y}} + f_{2} \left( t \right)\frac{\partial }{{\partial z}} + \frac{\partial }{{\partial t}} + \left[ { - \frac{1}{{2a_{1} a_{4} }}y\frac{{d^{2} }}{{dt^{2} }}f_{1} \left( t \right)} \right. \\ & \quad \left. { - \frac{1}{{2a_{1} a_{5} }}z\frac{{d^{2} }}{{dt^{2} }}f_{2} \left( t \right) + \frac{1}{{a_{1} }}\frac{d}{{dt}}f_{3} \left( t \right)} \right]\frac{\partial }{{\partial u}}. \\ \end{aligned}$$

3 $$\begin{aligned} X_{2} = & \,\left( {\frac{{ - 1}}{{2a_{4} }}y\frac{d}{{dt}}f_{4} \left( t \right) - \frac{1}{{2a_{5} }}z\frac{d}{{dt}}f_{5} \left( t \right) + f_{6} \left( t \right)} \right)\frac{\partial }{{\partial x}} \\ & \quad + \left( {z + f_{4} \left( t \right)} \right)\frac{\partial }{{\partial y}} + \left( {f_{5} \left( t \right) - \frac{{a_{5} }}{{a_{4} }}y} \right)\frac{\partial }{{\partial z}} + \left[ { - \frac{1}{{2a_{1} a_{4} }}y\frac{{d^{2} }}{{dt^{2} }}f_{4} \left( t \right)} \right. \\ & \quad \left. { - \frac{1}{{2a_{1} a_{5} }}z\frac{{d^{2} }}{{dt^{2} }}f_{5} \left( t \right) + \frac{1}{{a_{1} }}\frac{d}{{dt}}f_{6} \left( t \right)} \right]\frac{\partial }{{\partial u}}. \\ \end{aligned}$$

4 $$\begin{aligned} X_{3} = & \,\left( {\frac{x}{2} - \frac{1}{{2a_{4} }}y\frac{d}{{dt}}f_{7} \left( t \right) - \frac{1}{{2a_{5} }}z\frac{d}{{dt}}f_{8} \left( t \right) + f_{9} \left( t \right)} \right)\frac{\partial }{{\partial x}} \\ & \quad + \left( {y + f_{7} \left( t \right)} \right)\frac{\partial }{{\partial y}} + \left( {f_{8} \left( t \right) + z} \right)\frac{\partial }{{\partial z}} + \frac{3}{2}t\frac{\partial }{{\partial t}} \\ & \quad + \left[ { - \frac{1}{{2a_{1} a_{4} }}y\frac{{d^{2} }}{{dt^{2} }}f_{7} \left( t \right) - \frac{1}{{2a_{1} a_{5} }}z\frac{{d^{2} }}{{dt^{2} }}f_{8} \left( t \right) + \frac{1}{{a_{1} }}\frac{d}{{dt}}f_{9} \left( t \right) - a_{1} u - a_{3} } \right]\frac{\partial }{{\partial u}}. \\ \end{aligned}$$

Here, the (3 + 1)-dimensional nonlinear wave equation (NLWE) is considered, in which arbitrary functions appear within the Lie symmetry vectors. These functions are determined through an optimization process that aims to reduce the number of vectors involved in the commutator products. The commutator products of these vectors are given as follows:

5 $$\begin{aligned} \left[ {X_{1} ,X_{2} } \right] = & \,\left( {\frac{{ - 1}}{{2a_{4} }}f_{1} f_{4}^{'} - \frac{1}{{2a_{5} }}f_{2} f_{5}^{'} + f_{6}^{'} + \frac{1}{{2a_{4} }}f_{4} f_{1}^{'} + \frac{1}{{2a_{5} }}f_{5} f_{2}^{'} + ~y\left( {\frac{{ - 1}}{{2a_{4} }}f_{4}^{{''}} - \frac{1}{{2a_{4} }}f_{2}^{'} } \right)} \right. \\ & \quad \left. { + z\left( {\frac{{ - 1}}{{2a_{5} }}f_{5}^{{''}} + \frac{1}{{2a_{4} }}f_{1}^{'} } \right)} \right)\frac{\partial }{{\partial x}} + \left( {f_{2} + f_{4}^{'} } \right)\frac{\partial }{{\partial y}}\, + \,\left( {\frac{{ - a_{5} }}{{a_{4} }}f_{1} + f_{5}^{'} } \right)\frac{\partial }{{\partial z}} \\ & \quad + \left( {\frac{{ - 1}}{{2a_{4} a_{1} }}f_{4}^{{''}} f_{1} - \frac{1}{{2a_{5} a_{1} }}f_{5}^{{''}} f_{2} + \frac{1}{{a_{1} }}f_{6}^{{''}} + \frac{1}{{2a_{4} a_{1} }}f_{1}^{{''}} f_{4} + \frac{1}{{2a_{5} a_{1} }}f_{2}^{{''}} f_{5} } \right. \\ & \quad \left. { + z\left( {\frac{{ - 1}}{{2a_{5} a_{1} }}f_{5}^{{'''}} + \frac{1}{{2a_{4} a_{1} }}f_{1}^{{''}} } \right) + y\left( {\frac{{ - 1}}{{2a_{4} a_{1} }}f_{4}^{{'''}} - \frac{1}{{2a_{4} a_{1} }}f_{2}^{{''}} } \right)} \right)\frac{\partial }{{\partial u}}. \\ \end{aligned}$$

6 $$\begin{aligned} \left[ {X_{1} ,X_{3} } \right] = & \,\left( {\frac{{ - 1}}{{2a_{4} }}f_{1} f_{7}^{'} - \frac{1}{{2a_{5} }}f_{2} f_{8}^{'} + \frac{1}{2}f_{3} + f_{9}^{'} + \frac{1}{{2a_{4} }}f_{7} f_{1}^{'} + \frac{1}{{2a_{5} }}f_{8} f_{2}^{'} - \frac{3}{2}tf_{3}^{'} } \right. \\ & \quad \left. { + y\left( {\frac{1}{{4a_{4} }}f_{1}^{'} - \frac{1}{{2a_{4} }}f_{7}^{{''}} + \frac{3}{{4a_{4} }}tf_{1}^{{''}} } \right) + z\left( {\frac{1}{{4a_{5} }}f_{2}^{'} - \frac{1}{{2a_{5} }}f_{8}^{{''}} + \frac{3}{{4a_{5} }}tf_{2}^{{''}} } \right)} \right)\frac{\partial }{{\partial x}} \\ & \quad + \left( {f_{1} + f_{7}^{'} - \frac{3}{2}tf_{1}^{'} } \right)\frac{\partial }{{\partial y}} + \left( {f_{2} + f_{8}^{'} - \frac{3}{2}tf_{2}^{'} } \right)\frac{\partial }{{\partial z}} + \left( {\frac{{ - 1}}{{2a_{4} a_{1} }}f_{7}^{{''}} f_{1} - \frac{1}{{2a_{5} a_{1} }}f_{8}^{{''}} f_{2} } \right. \\ & \quad - \frac{3}{{2a_{1} }}tf_{3}^{{''}} - \frac{1}{{a_{1} }}f_{3}^{'} + \frac{1}{{a_{1} }}f_{9}^{{''}} + \frac{1}{{2a_{4} a_{1} }}f_{1}^{{''}} f_{7} + \frac{1}{{2a_{5} a_{1} }}f_{2}^{{''}} f_{8} + z\left( {\frac{3}{{4a_{5} a_{1} }}tf_{2}^{{'''}} + \frac{1}{{a_{5} a_{1} }}f_{2}^{{''}} } \right. \\ & \quad \left. { - \left( {\frac{3}{{4a_{5} a_{1} }}tf_{2}^{{\prime \prime \prime }} + \frac{1}{{a_{5} a_{1} }}f_{2}^{{''}} - \frac{1}{{2a_{4} a_{1} }}f_{8}^{{'''}} } \right) + y\left( {\frac{3}{{4a_{4} a_{1} }}tf_{1}^{{'''}} + \frac{1}{{a_{4} a_{1} }}f_{1}^{{''}} - \frac{1}{{2a_{4} a_{1} }}f_{7}^{{'''}} } \right)} \right)\frac{\partial }{{\partial u}}. \\ \end{aligned}$$

7 $$\begin{aligned} \left[ {X_{2} ,X_{3} } \right] = \, & \left( {\frac{{ - 1}}{{2a_{4} }}f_{4} f_{7} \prime - \frac{1}{{2a_{5} }}f_{5} f_{8} \prime + \frac{1}{2}f_{6} - \frac{3}{2}tf_{6} \prime + \frac{1}{{2a_{4} }}f_{7} f_{4} \prime } \right. \\ & \quad \left. { + \frac{1}{{2a_{5} }}f_{8} f_{5} \prime + y\left( {\frac{1}{{4a_{4} }}f_{4} \prime + \frac{1}{{2a_{4} }}f_{8} \prime + \frac{3}{{4a_{4} }}tf_{4}^{{\prime \prime }} } \right) + z\left( {\frac{1}{{4a_{5} }}f_{5} \prime - \frac{1}{{2a_{5} }}f_{7} \prime + \frac{3}{{4a_{5} }}tf_{5}^{{\prime \prime }} } \right)} \right)\frac{\partial }{{\partial x}} \\ & \quad + \left( {f_{4} - f_{8} - \frac{3}{2}tf_{4} \prime } \right)\frac{\partial }{{\partial y}} + \left( {f_{5} + \frac{{a_{5} }}{{a_{4} }}f_{7} - \frac{3}{2}tf_{5} \prime } \right)\frac{\partial }{{\partial z}} \\ & \quad + \left( {\frac{{ - 1}}{{2a_{4} a_{1} }}f_{7}^{{\prime \prime }} f_{4} - \frac{1}{{2a_{5} a_{1} }}f_{8}^{{\prime \prime }} f_{5} - \frac{3}{{2a_{1} }}tf_{6}^{{\prime \prime }} - \frac{1}{{a_{1} }}f_{6} \prime + \frac{1}{{2a_{4} a_{1} }}f_{4}^{{\prime \prime }} f_{7} } \right. \\ & \quad \left. { + \frac{1}{{2a_{5} a_{1} }}f_{5}^{{\prime \prime }} f_{8} + z\left( {\frac{3}{{4a_{5} a_{1} }}tf_{5}^{{\prime \prime \prime }} + \frac{1}{{a_{5} a_{1} }}f_{5}^{{\prime \prime }} - \frac{1}{{2a_{4} a_{1} }}f_{7}^{{\prime \prime }} } \right) + y\left( {\frac{3}{{4a_{4} a_{1} }}tf_{4}^{{\prime \prime \prime }} + \frac{1}{{a_{4} a_{1} }}f_{4}^{{\prime \prime }} + \frac{1}{{2a_{4} a_{1} }}f_{8}^{{\prime \prime }} } \right)} \right)\frac{\partial }{{\partial u}}. \\ \end{aligned}$$

These commutative products^51,53,62^ leads to a system of ordinary differential equations (ODEs) in ${f}_{i}(t$*)* in the following form:

8 $$\left. \begin{gathered} \frac{{ - 1}}{{2a_{4} }}f_{1} f_{4}^{{\text{'}}} - \frac{1}{{2a_{5} }}f_{2} f_{5}^{{\text{'}}} + f_{6}^{{\text{'}}} + \frac{1}{{2a_{4} }}f_{4} f_{1}^{{\text{'}}} \hfill \\ + \frac{1}{{2a_{5} }}f_{5} f_{2}^{{\text{'}}} = 0,{\text{~}}\frac{{ - 1}}{{2a_{4} }}f_{4}^{{{\text{''}}}} - \frac{1}{{2a_{4} }}f_{2}^{{\text{'}}} = 0, \hfill \\ \frac{{ - 1}}{{2a_{5} }}f_{5}^{{{\text{''}}}} + \frac{1}{{2a_{4} }}f_{1}^{{\text{'}}} = 0, \hfill \\ f_{2} + f_{4}^{{\text{'}}} = 0,{\text{~}}\frac{{ - a_{5} }}{{a_{4} }}f_{1} + f_{5}^{{\text{'}}} = 0, \hfill \\ \frac{{ - 1}}{{2a_{5} a_{1} }}f_{5}^{{{\text{'''}}}} + \frac{1}{{2a_{4} a_{1} }}f_{1}^{{{\text{''}}}} = 0, \hfill \\ \frac{{ - 1}}{{2a_{4} a_{1} }}f_{4}^{{{\text{''}}}} f_{1} - \frac{1}{{2a_{5} a_{1} }}f_{5}^{{{\text{''}}}} f_{2} + \frac{1}{{a_{1} }}f_{6}^{{{\text{''}}}} + \frac{1}{{2a_{4} a_{1} }}f_{1}^{{{\text{''}}}} f_{4} + \frac{1}{{2a_{5} a_{1} }}f_{2}^{{{\text{''}}}} f_{5} = 0, \hfill \\ \frac{{ - 1}}{{2a_{4} a_{1} }}f_{4}^{{{\text{'''}}}} - \frac{1}{{2a_{4} a_{1} }}f_{2}^{{{\text{''}}}} = 0, \hfill \\ \frac{{ - 1}}{{2a_{4} }}f_{1} f_{7}^{{\text{'}}} - \frac{1}{{2a_{5} }}f_{2} f_{8}^{{\text{'}}} - f_{3} + f_{9}^{{\text{'}}} + \frac{1}{{2a_{4} }}f_{7} f_{1}^{{\text{'}}} + \frac{1}{{2a_{5} }}f_{8} f_{2}^{{\text{'}}} - \frac{3}{2}tf_{3}^{{\text{'}}} = 0, \hfill \\ \frac{1}{{a_{4} }}f_{1}^{{\text{'}}} - \frac{1}{{2a_{4} }}f_{7}^{{{\text{''}}}} + \frac{3}{{4a_{4} }}tf_{1}^{{{\text{''}}}} = 0, \hfill \\ \frac{1}{{a_{5} }}f_{2}^{{\text{'}}} - \frac{1}{{2a_{5} }}f_{8}^{{{\text{''}}}} + \frac{3}{{4a_{5} }}tf_{2}^{{{\text{''}}}} = 0, \hfill \\ 2f_{7}^{{\text{'}}} - f_{1} - 3tf_{1}^{{\text{'}}} = 0, \hfill \\ 2f_{8}^{{\text{'}}} - f_{2} - 3tf_{2}^{{\text{'}}} = 0, \hfill \\ \frac{{ - 1}}{{2a_{4} a_{1} }}f_{7}^{{{\text{''}}}} f_{1} - \frac{1}{{2a_{5} a_{1} }}f_{8}^{{{\text{''}}}} f_{2} - \frac{3}{{2a_{1} }}tf_{3}^{{{\text{''}}}} - \frac{5}{{2a_{1} }}f_{3}^{{\text{'}}} \hfill \\ + \frac{1}{{a_{1} }}f_{9}^{{{\text{''}}}} + \frac{1}{{2a_{4} a_{1} }}f_{1}^{{{\text{''}}}} f_{7} + \frac{1}{{2a_{5} a_{1} }}f_{2}^{{{\text{''}}}} f_{8} = 0, \hfill \\ \frac{3}{{4a_{5} a_{1} }}tf_{2}^{{{\text{'''}}}} + \frac{7}{{4a_{5} a_{1} }}f_{2}^{{{\text{''}}}} - \frac{1}{{2a_{4} a_{1} }}f_{8}^{{{\text{'''}}}} = 0, \hfill \\ \frac{3}{{4a_{4} a_{1} }}tf_{1}^{{{\text{'''}}}} + \frac{7}{{4a_{4} a_{1} }}f_{1}^{{{\text{''}}}} - \frac{1}{{2a_{4} a_{1} }}f_{7}^{{{\text{'''}}}} = 0, \hfill \\ \frac{{ - 1}}{{2a_{4} }}f_{4} f_{7}^{{\text{'}}} - \frac{1}{{2a_{5} }}f_{5} f_{8}^{{\text{'}}} + \frac{1}{2}f_{6} - \frac{3}{2}tf_{6}^{{\text{'}}} + \frac{1}{{2a_{4} }}f_{7} f_{4}^{{\text{'}}} + \frac{1}{{2a_{5} }}f_{8} f_{5}^{{\text{'}}} = 0, \hfill \\ \frac{1}{{4a_{4} }}f_{4}^{{\text{'}}} + \frac{1}{{2a_{4} }}f_{8}^{{\text{'}}} + \frac{3}{{4a_{4} }}tf_{4}^{{{\text{''}}}} = 0, \hfill \\ \frac{1}{{4a_{5} }}f_{5}^{{\text{'}}} - \frac{1}{{2a_{5} }}f_{7}^{{\text{'}}} + \frac{3}{{4a_{5} }}tf_{5}^{{{\text{''}}}} = 0, \hfill \\ f_{4} - f_{8} - \frac{3}{2}tf_{4}^{{\text{'}}} = 0, \hfill \\ f_{5} + \frac{{a_{5} }}{{a_{4} }}f_{7} - \frac{3}{2}tf_{5}^{{\text{'}}} = 0, \hfill \\ \frac{{ - 1}}{{2a_{4} a_{1} }}f_{7}^{{{\text{''}}}} f_{4} - \frac{1}{{2a_{5} a_{1} }}f_{8}^{{{\text{''}}}} f_{5} - \frac{3}{{2a_{1} }}tf_{6}^{{{\text{''}}}} - \frac{1}{{a_{1} }}f_{6}^{{\text{'}}} \hfill \\ + \frac{1}{{2a_{4} a_{1} }}f_{4}^{{{\text{''}}}} f_{7} + \frac{1}{{2a_{5} a_{1} }}f_{5}^{{{\text{''}}}} f_{8} = 0, \hfill \\ \frac{3}{{4a_{5} a_{1} }}tf_{5}^{{{\text{'''}}}} + \frac{1}{{a_{5} a_{1} }}f_{5}^{{{\text{''}}}} - \frac{1}{{2a_{4} a_{1} }}f_{7}^{{{\text{''}}}} = 0, \hfill \\ \frac{3}{{4a_{4} a_{1} }}tf_{4}^{{{\text{'''}}}} + \frac{1}{{a_{4} a_{1} }}f_{4}^{{{\text{''}}}} + \frac{1}{{2a_{4} a_{1} }}f_{8}^{{{\text{''}}}} = 0. \hfill \\ \end{gathered} \right\}$$

This system of differential equations has infinite number of solutions. One of the obtained results are given by the following eighteen vectors.

$${X}_{1}=\frac{\partial }{\partial y}+\frac{\partial }{\partial t}, {X}_{2}=\frac{\partial }{\partial z}+\frac{\partial }{\partial t}, {X}_{3}=\frac{\partial }{\partial x}+\frac{\partial }{\partial t} , {X}_{4}=t\frac{\partial }{\partial x}+\frac{\partial }{\partial t}+\frac{\partial }{\partial u},$$

$${X}_{5}=\frac{\partial }{\partial x}+z\frac{\partial }{\partial y}-y\frac{\partial }{\partial z}, {X}_{6}=t\frac{\partial }{\partial x}+z\frac{\partial }{\partial y}-y\frac{\partial }{\partial z}+\frac{\partial }{\partial u}, {X}_{7}=z\frac{\partial }{\partial y}+\left(1-y\right)\frac{\partial }{\partial z},$$

$${X}_{8}=-\frac{y}{2}\frac{\partial }{\partial x}+t\frac{\partial }{\partial y}+\frac{\partial }{\partial t}, { X}_{9}=-\frac{z}{2}\frac{\partial }{\partial x}+t\frac{\partial }{\partial z}+\frac{\partial }{\partial t}, {X}_{10}=\left(z+1\right)\frac{\partial }{\partial y}-y\frac{\partial }{\partial z},$$

$${X}_{11}=-\frac{z}{2}\frac{\partial }{\partial x}+z\frac{\partial }{\partial y}+\left(t-y\right)\frac{\partial }{\partial z}, {X}_{12}=-\frac{y}{2}\frac{\partial }{\partial x}+\left(t+z\right)\frac{\partial }{\partial y}-y\frac{\partial }{\partial z},$$

$${X}_{13}=\left(t+\frac{x}{2}\right)\frac{\partial }{\partial x}+y\frac{\partial }{\partial y}+z\frac{\partial }{\partial z}+\frac{3t}{2}\frac{\partial }{\partial t}-u\frac{\partial }{\partial u},$$

$${X}_{14}=\left(1+\frac{x}{2}\right)\frac{\partial }{\partial x}+y\frac{\partial }{\partial y}+z\frac{\partial }{\partial z}+\frac{3t}{2}\frac{\partial }{\partial t}-\left(u+1\right)\frac{\partial }{\partial u},$$

$${X}_{15}=\left(\frac{x}{2}\right)\frac{\partial }{\partial x}+\left(y+1\right)\frac{\partial }{\partial y}+z\frac{\partial }{\partial z}+\frac{3t}{2}\frac{\partial }{\partial t}-\left(u+1\right)\frac{\partial }{\partial u} ,$$

$${X}_{16}=\left(\frac{x}{2}\right)\frac{\partial }{\partial x}+y\frac{\partial }{\partial y}+\left(z+1\right)\frac{\partial }{\partial z}+\frac{3t}{2}\frac{\partial }{\partial t}-\left(u+1\right)\frac{\partial }{\partial u} ,$$

$${X}_{17}=\left(\frac{x-y}{2}\right)\frac{\partial }{\partial x}+\left(y+t\right)\frac{\partial }{\partial y}+z\frac{\partial }{\partial z}+\frac{3t}{2}\frac{\partial }{\partial t}-\left(u+1\right)\frac{\partial }{\partial u},$$

9 $${X}_{18}=\left(\frac{x-z}{2}\right)\frac{\partial }{\partial x}+y\frac{\partial }{\partial y}+\left(z+t\right)\frac{\partial }{\partial z}+\frac{3t}{2}\frac{\partial }{\partial t}-\left(u+1\right)\frac{\partial }{\partial u} .$$

## Optimal system of lie vectors

Lie vectors are mathematically defined as the infinitesimal generators of a Lie group, enabling the precise characterization of small transformations within the group. They play a fundamental role in the study of Lie groups and their applications across various fields, including differential geometry, physics, and robotics. An appropriate selection of Lie vectors is essential for accurately describing group transformations and for simplifying computations in these areas. By carefully selecting suitable Lie vectors, one can significantly improve both the computational efficiency and the accuracy of algorithms that rely on Lie theory. To this end, systematic procedures based on the concepts of commutation relations, Eq. (10), and the adjoint representation, Eq. (11), are employed to identify the optimal system of vectors, including single, double, triple, and quadruple linear combinations.

10 $$\left[{X}_{i},{X}_{j}\right]={X}_{i}{X}_{j}-{X}_{j}{X}_{i}.$$

11 $$Ad(\mathit{exp}(\varepsilon v)){w}_{0}={\sum }_{n=0}^{\infty }\frac{{\varepsilon }^{n}}{n!}(adv{)}^{n}({w}_{0})={w}_{0}-\varepsilon [\text{v,}{\text{w}}_{0}]+\frac{{\varepsilon }^{2}}{2}[v,[\text{v,}{\text{w}}_{0}]]-\dots$$

To avoid much mathematical analysis, more details about commutator tables, adjoint matrix, and optimal system representation can be found in^51,53^.

### Reduction using single vectors

The optimization procedure leads to the following optimal single vectors: ${X}_{6},{X}_{7},{X}_{9},{X}_{10},{X}_{12},{X}_{13},{X}_{16} and {X}_{18}$. Moreover, the dual combinations were found to be ${X}_{6}+{X}_{10}, { X}_{7}+{X}_{10}, {X}_{10}+{X}_{12}, { X}_{7}+{X}_{16}, { X}_{9}+{X}_{16}, {X}_{13}+{X}_{16}$,${X}_{13}+{X}_{18}$, the triple combinations were defined as X_6_ + X_7_ + X_10_, X_6_ + X_7_ + X_12_, X_6_ + X_10_ + X_12_, + X_7_ + X_10_ + X_12_, X_9_ + X_13_ + X_18_, X_13_ + X_16_ + X_18_. Moreover, only one quadruple combination is obtained after considering the simplification of adjoint matrices^51,53^. This combination is ${X}_{12}+{X}_{13}+{X}_{16}+{X}_{18}$. These vectors are used sequentially to detect exact solutions of NLWE^63–66^.

### ***Case 1: using***${{\varvec{X}}}_{6}$

This vector is used to reduce the number of independent variables of Eq. (1) and transform it to be:

12 $$p{w}_{rrrr}+\left(p+{q}^{2}+pw\right){w}_{rr}+4p\left(p{w}_{pp}+\frac{1}{4}{w}_{r}^{2}+{w}_{p}+\frac{1}{4}{w}_{rq}\right)=0,$$

where, $$p={y}^{2}+{z}^{2}, q=t, r=-t\text{arctan}\left(\frac{y}{z}\right)+x, w=u-\text{arctan}\left(\frac{y}{z}\right)$$.

Now, Eq. (12) is tested for Lie vectors using MAPLE to obtain one of its infinitesimal vectors in the form, $Y=q\frac{\partial }{\partial r}+\frac{\partial }{\partial w}$. This vector is employed to reduce the Eq. (12) into:

13 $$4s{v}_{ss}+4{v}_{s}=0.$$

Where, $o=q,s=p, v\left(s,o\right)=w\left(p,q,r\right)-\frac{r}{q}$. This equation has an exact solution in the form:

14 $$v\left(s,o\right)={{F}_{1}\left(o\right)+F}_{2}\left(o\right)\text{ln}\left(s\right).$$

where, ${F}_{1}$ and ${F}_{2}$ are arbitrary functions in their arguments.

Finally, the solution of Eq. (1) can be formulated in the form:

15 $${u}_{1}\left(x,y,z,t\right)=\text{arctan}\left(\frac{y}{z}\right)+{F}_{2}\left(t\right)\text{ln}\left({y}^{2}+{z}^{2}\right)+{F}_{1}\left(t\right).$$

**Fig. 1:**
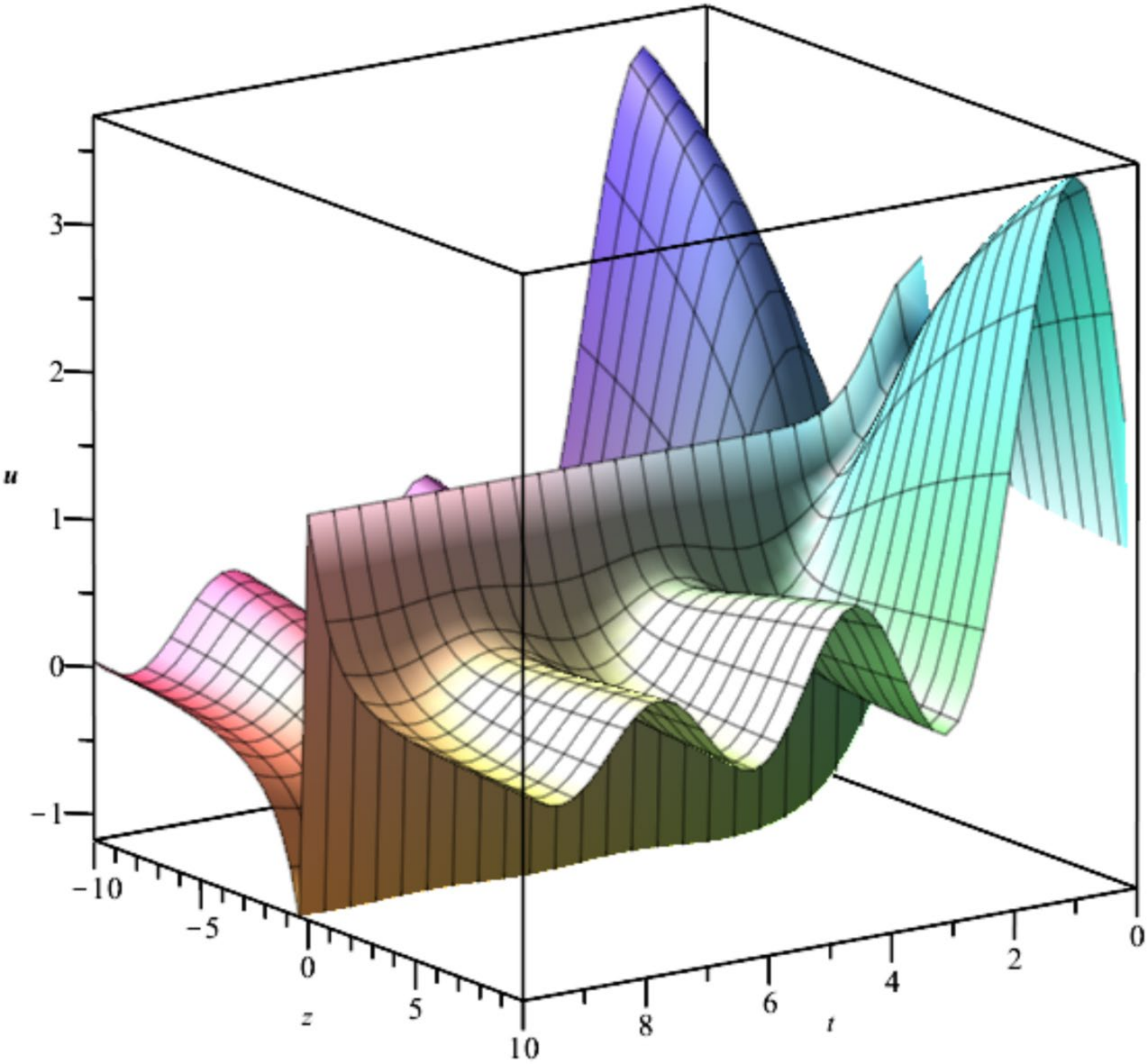
Illustration of ${u}_{1}\left(x,y,z,t\right)$ for ${F}_{1}\left(t\right)={e}^{-{t}^{2}},{F}_{2}\left(t\right)=\frac{{\text{sin}}^{2}\left(t\right)}{t}, y=1.$

The behavior of the gas bubbles is depicted in Fig. 1 for ${F}_{1}\left(t\right)={e}^{-{t}^{2}},{F}_{2}\left(t\right)=\frac{{\text{sin}}^{2}\left(t\right)}{t}$.

### ***Case 2: using***${{\varvec{X}}}_{7}$

This vector is used to reduce the number of independent variables in Eq. (1), resulting in:

16 $${w}_{pppp}+\left(1+w\right){w}_{pp}+\left(4+4q\right){w}_{qq}+{w}_{p}^{2}+4{w}_{q}+{w}_{pr}=0,$$

Where, $$p=x,q={y}^{2}+{z}^{2}-2y, r=t, w\left(p,q,r\right)=u\left(x,y,z,t\right).$$

This equation has Lie infinitesimal vectors. One of these vectors is $$\left( {1 + \frac{p}{4}} \right)\frac{\partial }{{\partial p}} + \left( {q + 1} \right)\frac{\partial }{{\partial q}}$$$$+ \left( {\frac{{3r}}{4}} \right)\frac{\partial }{{\partial r}}$$$$- \left( {\frac{{w + 1}}{2}} \right)\frac{\partial }{{\partial w}}$$.

The invariant transformation is defined by:

$$o=\frac{r}{{\left(p+4\right)}^{3}} , s=\frac{q+1}{{p}^{4}+16{p}^{3}+96{p}^{2}+256p+256},$$

17 $$v\left(s,o\right)={p}^{2}w\left(p,q,r\right)+{p}^{2}+8pw\left(p,q,r\right)+8p+16w\left(p,q,r\right).$$

This transformation transforms Eq. (16) into:

18 $$\begin{aligned} o^{4} v_{{oooo}} & + \frac{{16}}{3}o^{3} sv_{{ooos}} + \frac{{32}}{3}o^{2} s^{2} v_{{ooss}} + \frac{{256}}{{27}}os^{3} v_{{osss}} + \frac{{256}}{{81}}s^{4} v_{{ssss}} + \frac{{32}}{3}o^{3} v_{{ooo}} \\ & + \frac{{136}}{3}o^{2} sv_{{oos}} + 64os^{2} v_{{oss}} + \frac{{2432}}{{81}}s^{3} v_{{sss}} + \frac{o}{9}\left( {ov + 276o - \frac{1}{3}} \right)v_{{oo}} \\ & + \frac{{8s}}{{27}}\left( {ov + 320o - \frac{1}{6}} \right)v_{{os}} + \frac{{16s}}{{81}}\left( {sv + 367s + \frac{1}{4}} \right)v_{{ss}} + \frac{{o^{2} }}{9}v_{o}^{2} + \left( { - \frac{5}{{81}} + \frac{4}{9}ov + \frac{{8os}}{{27}}v_{s} + \frac{{712o}}{{27}}} \right)v_{o} \\ & + \frac{{16s^{2} }}{{81}}v_{s}^{2} + \left( {\frac{4}{{81}} + \frac{{52s}}{{81}}v + \frac{{3736s}}{{81}}} \right)v_{s} + \frac{{10}}{{81}}\left( {28 + v} \right)\left( {16 + v} \right) = 0. \\ \end{aligned}$$

Equation (18) has a Lie vector in the form; $(s{o}^\frac{1}{3})\frac{\partial }{\partial s}+\left(\frac{3}{4}{o}^\frac{4}{3}\right)\frac{\partial }{\partial o}-\left(\frac{1+o\left(6v+96\right)}{12{o}^\frac{2}{3}}\right)\frac{\partial }{\partial v}$ which transforms the equation to:

19 $$81{\alpha }^{4}{\phi }_{\alpha \alpha }+189{\alpha }^{3}{\phi }_{\alpha }+36{\alpha }^{2}\phi -8=0,$$

where, $$\alpha =o{s}^{-\frac{3}{4}}, \phi \left(\alpha \right)=\sqrt{s} \left(-\frac{1}{3o}+v\left(s,o\right)+16\right).$$

Now, Eq. (19) could be solved analytically. Using Back substitutions, the solution of Eq. (1) is formulated in the form:

20 $$\begin{aligned} u_{2} \left( {x,y,z,t} \right) = & \,\frac{1}{{36t^{{\frac{8}{3}}} }}\left( {36C_{1} t^{2} {\text{ln}}\left( t \right) - 27C_{1} t^{2} ln\left( {y^{2} + z^{2} - 2y + 1} \right)} \right) \\ & \quad - 36t^{{\frac{8}{3}}} + 12xt^{{\frac{5}{3}}} + 2y^{2} t^{{\frac{2}{3}}} + 2z^{2} t^{{\frac{2}{3}}} + 36C_{2} t^{2} + 48t^{{\frac{5}{3}}} - 4yt^{{\frac{2}{3}}} + 2t^{{\frac{2}{3}}} . \\ \end{aligned}$$

**Fig. 2:**
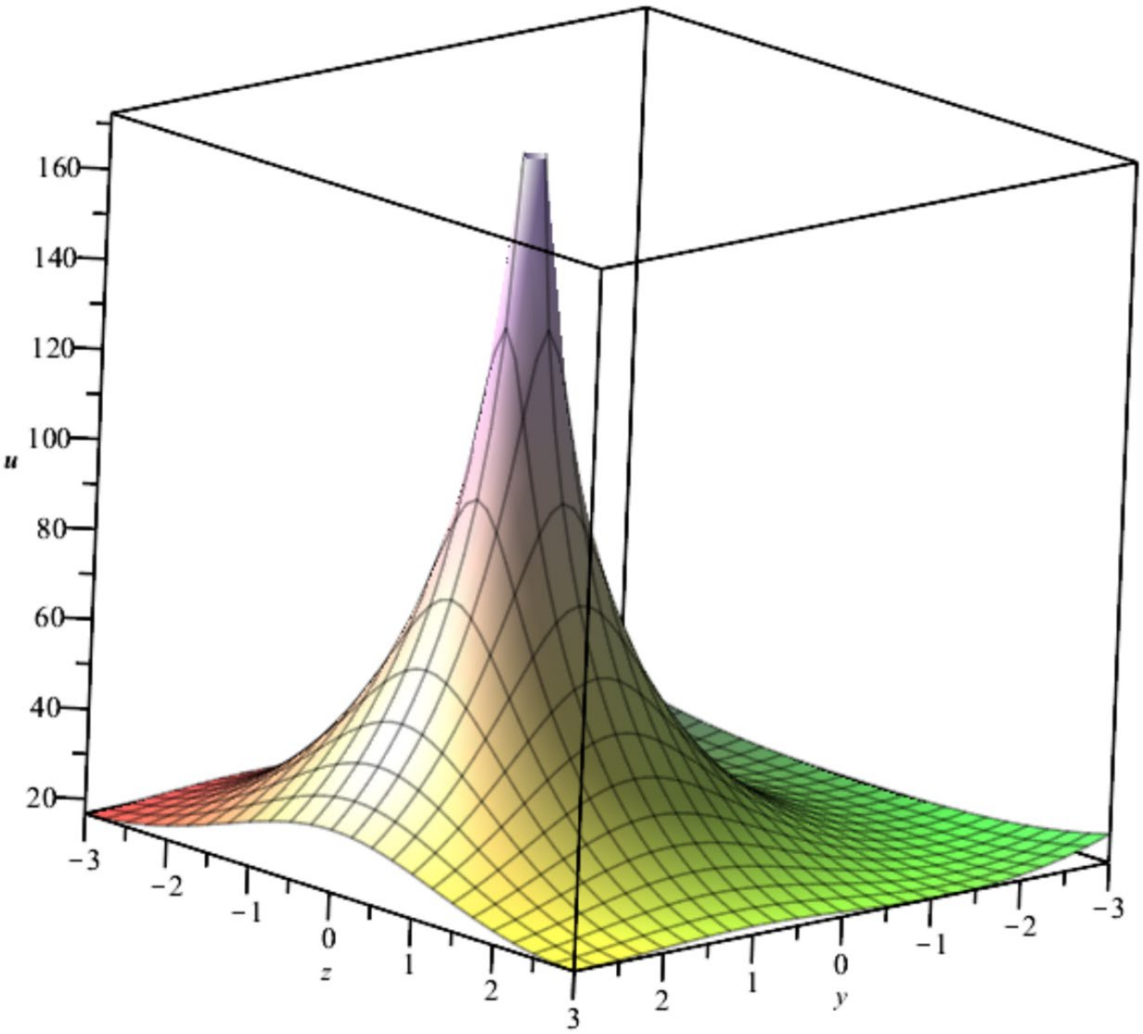
Illustration of ${u}_{2}\left(x,y,z,t\right)$ at $x=1, t=1, {C}_{1}={C}_{2}=1$.

The bubble dynamics is illustrated in Fig. 2

### ***Case 3: using***${{\varvec{X}}}_{9}$

Similar procedures are being used to extract and create new families of solutions for NLWE in its three-dimensional form. The vector ${X}_{9}$ transforms Eq. (1) into:

21 $${w}_{rrrr}+\left(1+w-\frac{q}{4}\right){w}_{rr}+{w}_{r}^{2}+{w}_{pp}+4{w}_{qq}=0,$$

where, $$p=y, q={t}^{2}-2z, r=\frac{1}{6}\left(3tz-{t}^{3}\right)+x, w\left(p,q,r\right)=u\left(x,y,z,t\right).$$

At this stage, Eq. (21) undergoes the Lie infinitesimal test, which yields a number of infinitesimal generators. These generators, along with their associated reduction procedures, are summarized in Table 1.

**Table 1:** Lie Infinitesimal Vectors of Eq. (21) Using ${\text{X}}_{9}$.

Case 3.1	Vector	$\frac{\partial }{\partial r}$
	Reduced Form	${v}_{ss}+4{v}_{oo}=0$, Where,$o=q,s=p,v\left(s,o\right)=w\left(p,q,r\right).$
	Solution	$v\left(s,o\right)={F}_{1}\left(o-2Is\right)+{F}_{2}\left(o+2Is\right)$
	Final Solution of (1)	${u}_{3}={F}_{1}\left({t}^{2}-2z-2Iy\right)+{F}_{2}\left({t}^{2}-2z+2Iy\right).$
Case 3.2	Vector	$\frac{\partial }{\partial q}+\frac{1}{4}\frac{\partial }{\partial w}$
	Reduced Form	${v}_{o}^{2}+{v}_{ss}+\left(1+v\right){v}_{oo}+{v}_{oooo}=0$, Where,$o=r,s=p,v\left(s,o\right)=w\left(p,q,r\right)-\frac{q}{4}.$
	Solution	$v\left(s,o\right)=-12{C}_{3}^{2}{\text{tanh}}^{2}\left({C}_{2}s+{C}_{3}o+{C}_{1}\right)-\frac{{C}_{2}^{2}+{C}_{3}^{2}-8{C}_{3}^{4}}{{C}_{3}^{2}}$
	Final Solution of (1)	${u}_{4}={t}^{2}-\frac{z}{2}-12{C}_{3}^{2}{\text{tanh}}^{2}\left({C}_{1}+{C}_{2}y+{C}_{3}\left(x+\frac{3tz-{t}^{3}}{6}\right)\right)-\frac{{C}_{2}^{2}+{C}_{3}^{2}-8{C}_{3}^{4}}{{C}_{3}^{2}}.$
Case 3.3	Vector	$q\frac{\partial }{\partial p}-4p\frac{\partial }{\partial q}-p\frac{\partial }{\partial w}.$
	Reduced Form 1	$v{v}_{oo}+{v}_{o}^{2}+16s{v}_{ss}+{v}_{oooo}+{v}_{oo}+16{v}_{s}=0$, Where, $o=r, s=4{p}^{2}+{q}^{2}, v=w-\frac{q}{4}$
	New level vector	$\frac{\partial }{\partial o}$
	Reduced Form 2	$\alpha {\phi }_{\alpha \alpha }+{\phi }_{\alpha }=0$, Where, $\alpha =s,\phi =v$
	Solution	$\phi ={C}_{1}+{C}_{2}\text{ln}\left(\alpha \right).$
	Final Solution of (1)	${u}_{5}={C}_{1}+\frac{{t}^{2}}{4}-\frac{z}{2}+{C}_{2}\text{ln}\left(4{y}^{2}+{\left({t}^{2}-2z\right)}^{2}\right).$

**Fig. 3:**
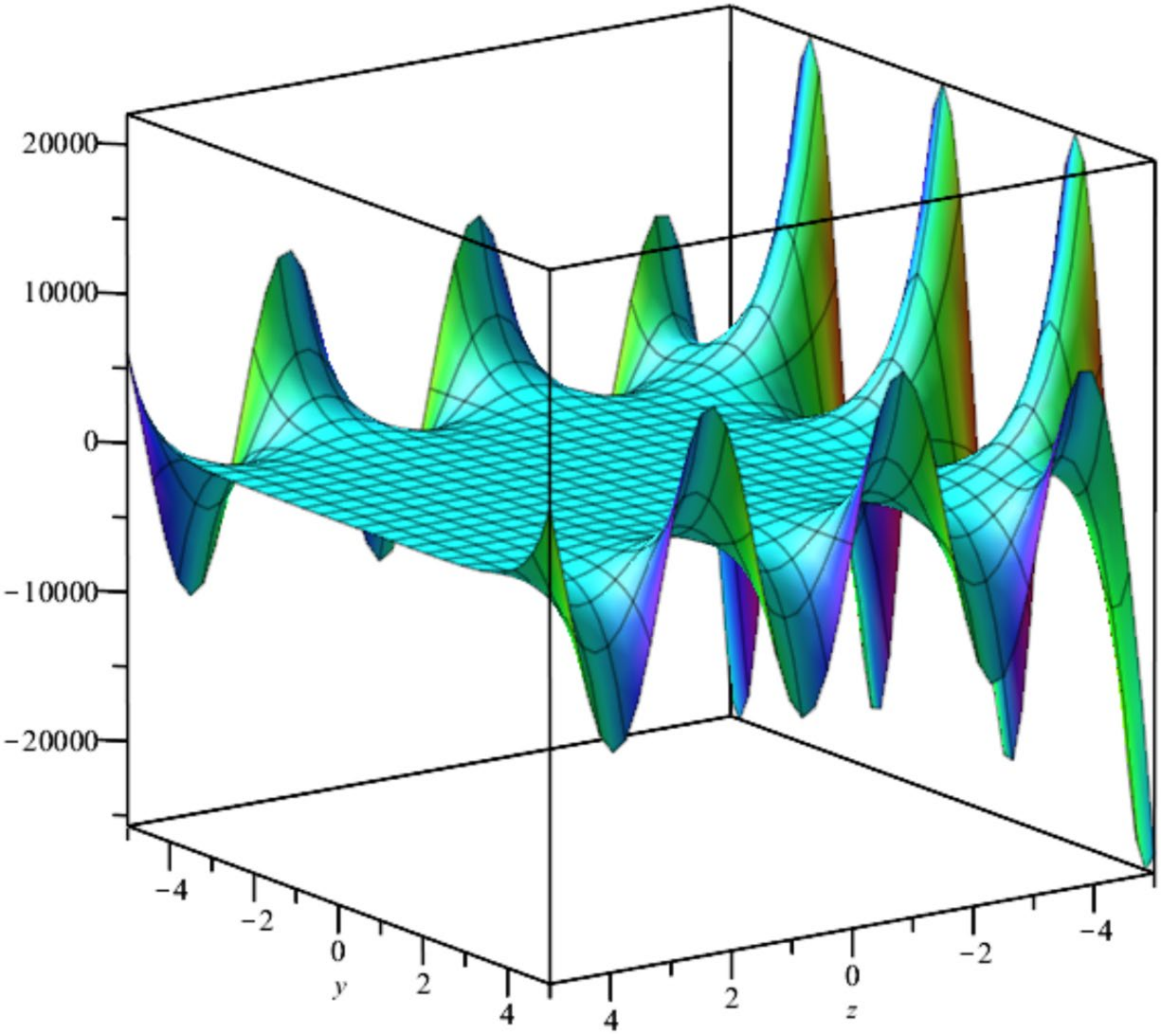
Illustration of ${u}_{3}$ at $t=0$ For ${F}_{1}=\text{exp}\left({t}^{2}-2z-2Iy\right), {F}_{2}=sin\left({t}^{2}-2z+2Iy\right)$.

**Fig. 4:**
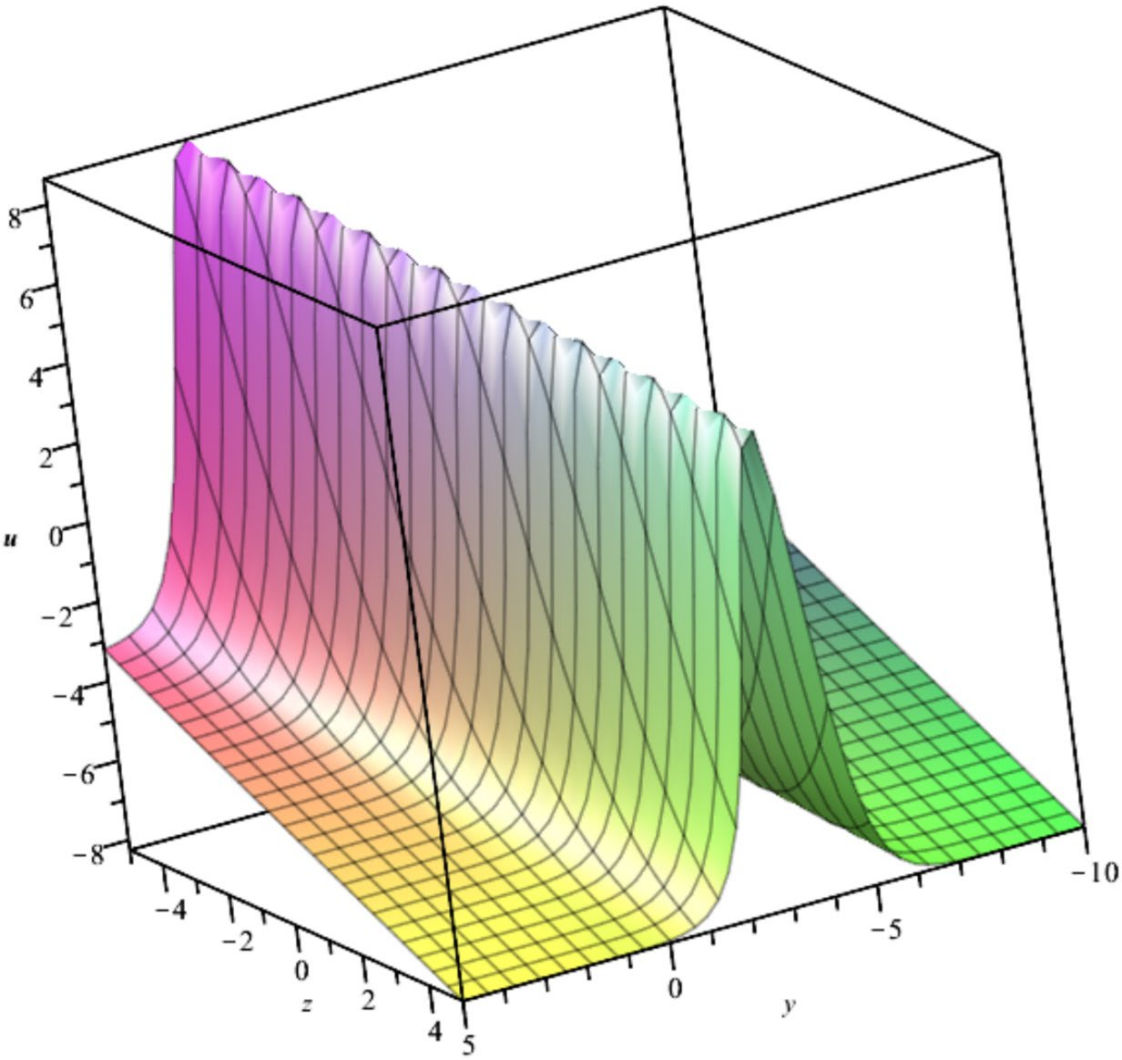
Illustration of ${u}_{4}$ at ${C}_{1}={C}_{2}={C}_{3}=1, x=0, t=1$.

**Fig. 5:**
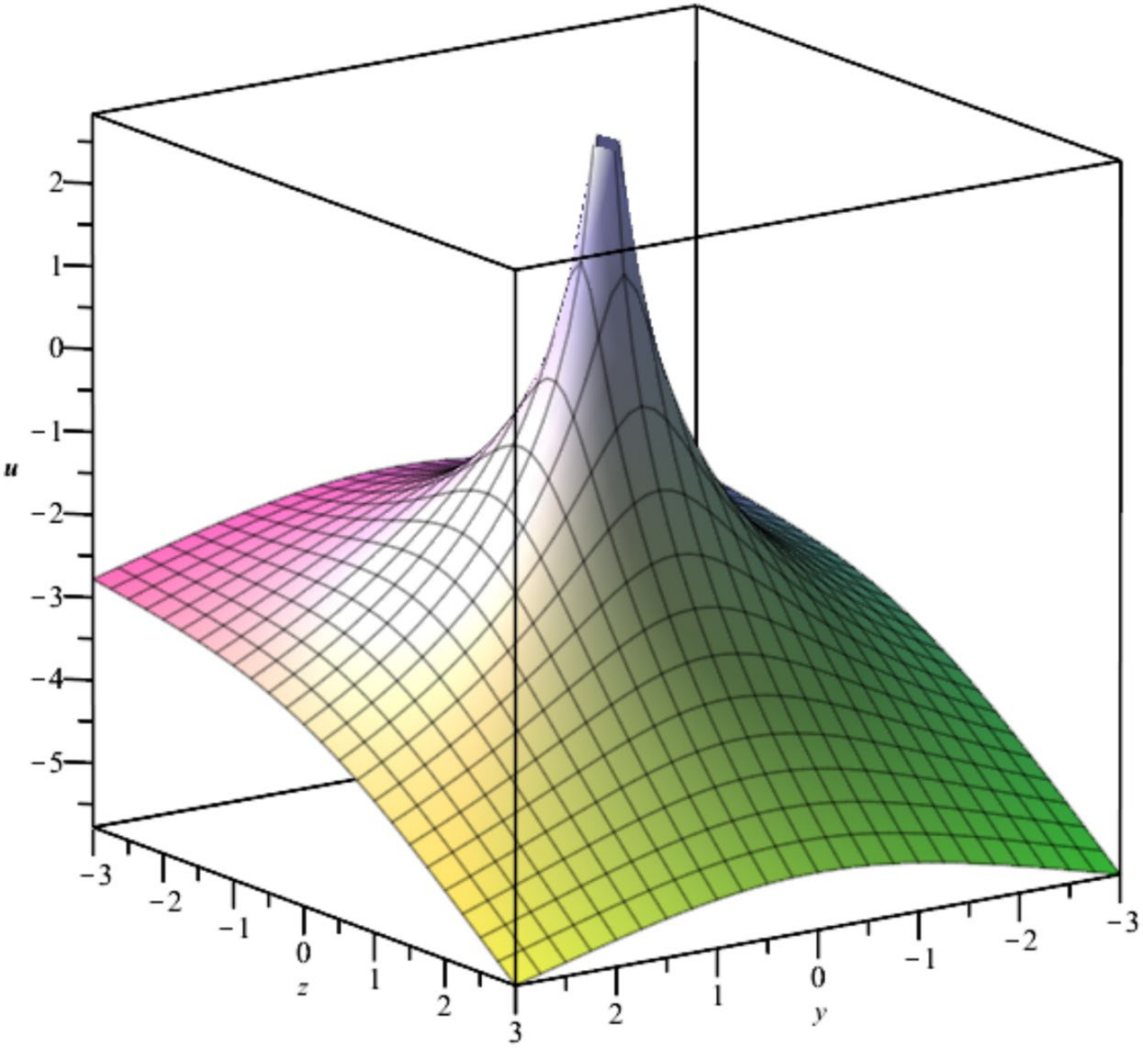
Illustration of ${u}_{5}$ at ${C}_{1}=0,{C}_{2}=-1,t=0$.

The solutions, ${u}_{3},{u}_{4}and {u}_{5}$ are illustrated in Figs. 3, 4 and 5.

### ***Case 4: using***${{\varvec{X}}}_{10}$

This vector transforms Eq. (1) to:

22 $${w}_{p}^{2}+w{w}_{pp}-2q{w}_{qq}+{w}_{pr}+{w}_{pppp}+{w}_{pp}+{w}_{qq}-2{w}_{q}=0,$$

where, $$p=x,q=-\left(\frac{{z}^{2}+{y}^{2}}{2}\right)-z, r=t, w=u$$.

Equation (22) has an infinitesimal vector, $\left(\frac{p}{2}+1\right)\frac{\partial }{\partial p}+\left(2q-1\right)\frac{\partial }{\partial q}+\frac{3r}{2}\frac{\partial }{\partial r}-\left(w+1\right)\frac{\partial }{\partial w}$.

Following the same procedures, the following solution can be obtained.

23 $$\begin{aligned} u_{6} = & \,\frac{1}{{36t^{{\frac{{14}}{3}}} }}\left( { - 27C_{1} t^{4} {\text{ln}}\left( { - y^{2} - z^{2} - 2z - 1} \right) + t^{{\frac{8}{3}}} \left( {2y^{2} + 2z^{2} + 4z + 2} \right)} \right. \\ & \quad \left. { + 27C_{1} {\text{ln}}\left( 2 \right)t^{4} + 36C_{1} t^{4} {\text{ln}}\left( t \right) - 36t^{{\frac{{14}}{3}}} + 36C_{2} t^{4} + t^{{\frac{{11}}{3}}} \left( {24 + 12x} \right)} \right). \\ \end{aligned}$$

**Fig. 6:**
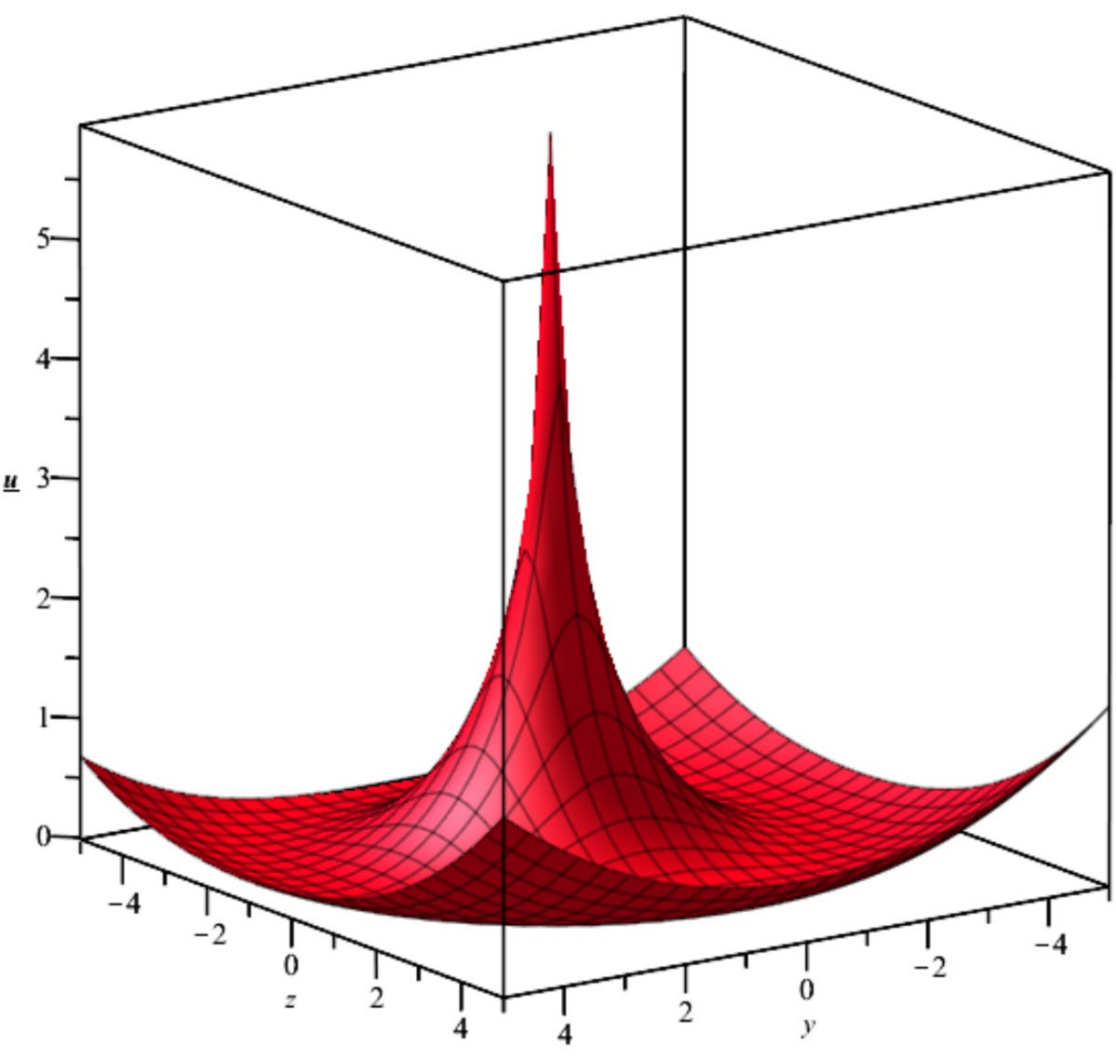
Illustration of ${u}_{6}$ at ${C}_{1}={C}_{2}=1,x=0, t=1$.

The behavior of the solution described by (23) is depicted hereafter in Fig. 6.

### ***Case 5: using***${{\varvec{X}}}_{12}$

Using the invariant variables, P = z − 2x, q = t, r = 4x (z−2x) +4tx + 4x^2^ + y^2^, w (p, q, r) = u (x, y, z, t).

the following solution is obtained.

24 $${u}_{7}={C}_{1}-\frac{t}{2}+{C}_{2}\text{ln}\left({t}^{2}+2tz+{y}^{2}+{z}^{2}\right).$$

**Fig. 7:**
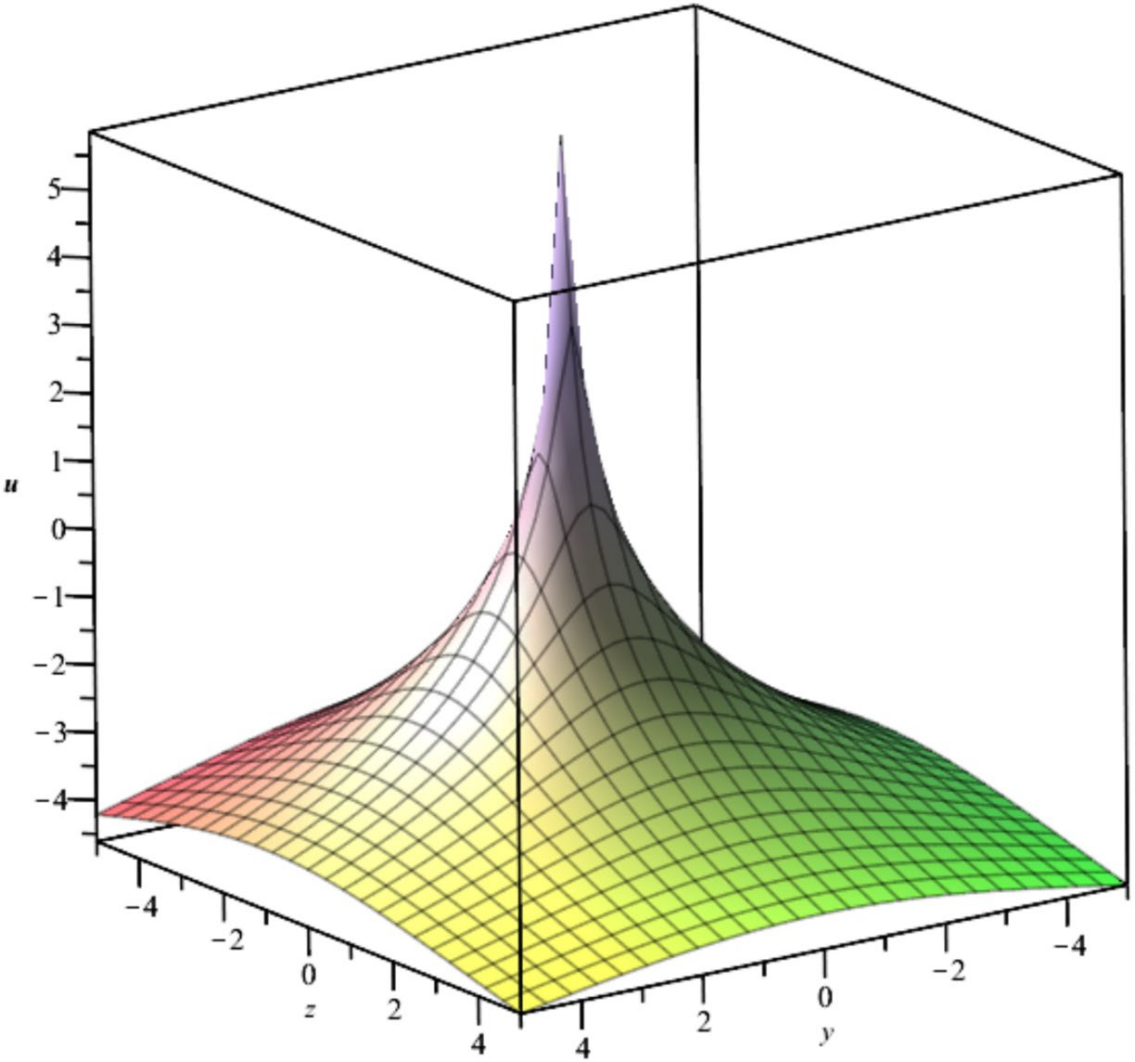
Illustration of ${u}_{7}$ at ${C}_{1}=0,{C}_{2}=-1, t=1$.

The behavior of the gas bubble due to the case in Eq. (24) is illustrated in Fig. 7

### ***Case 6: using***${{\varvec{X}}}_{13}$

The invariant variables of ${X}_{13}$ are $p=\frac{z}{y},q=t{y}^{-\frac{3}{2}},r=\frac{x-t}{\sqrt{y}}, w=yu$. The transformed equation is given by:

25 $$\begin{aligned} 6rqw_{{qr}} & + r^{2} w_{{rr}} + 9q^{2} w_{{qq}} + 12pqw_{{pq}} + 4rpw_{{pr}} \\ & + 4p^{2} w_{{pp}} + 4w_{r}^{2} + 7rw_{r} + 4ww_{{rr}} + 27qw_{q} \\ & + 16pw_{p} + 4w_{{qr}} + 4w_{{rrrr}} + 8w + 4w_{{pp}} = 0. \\ \end{aligned}$$

**Fig. 8:**
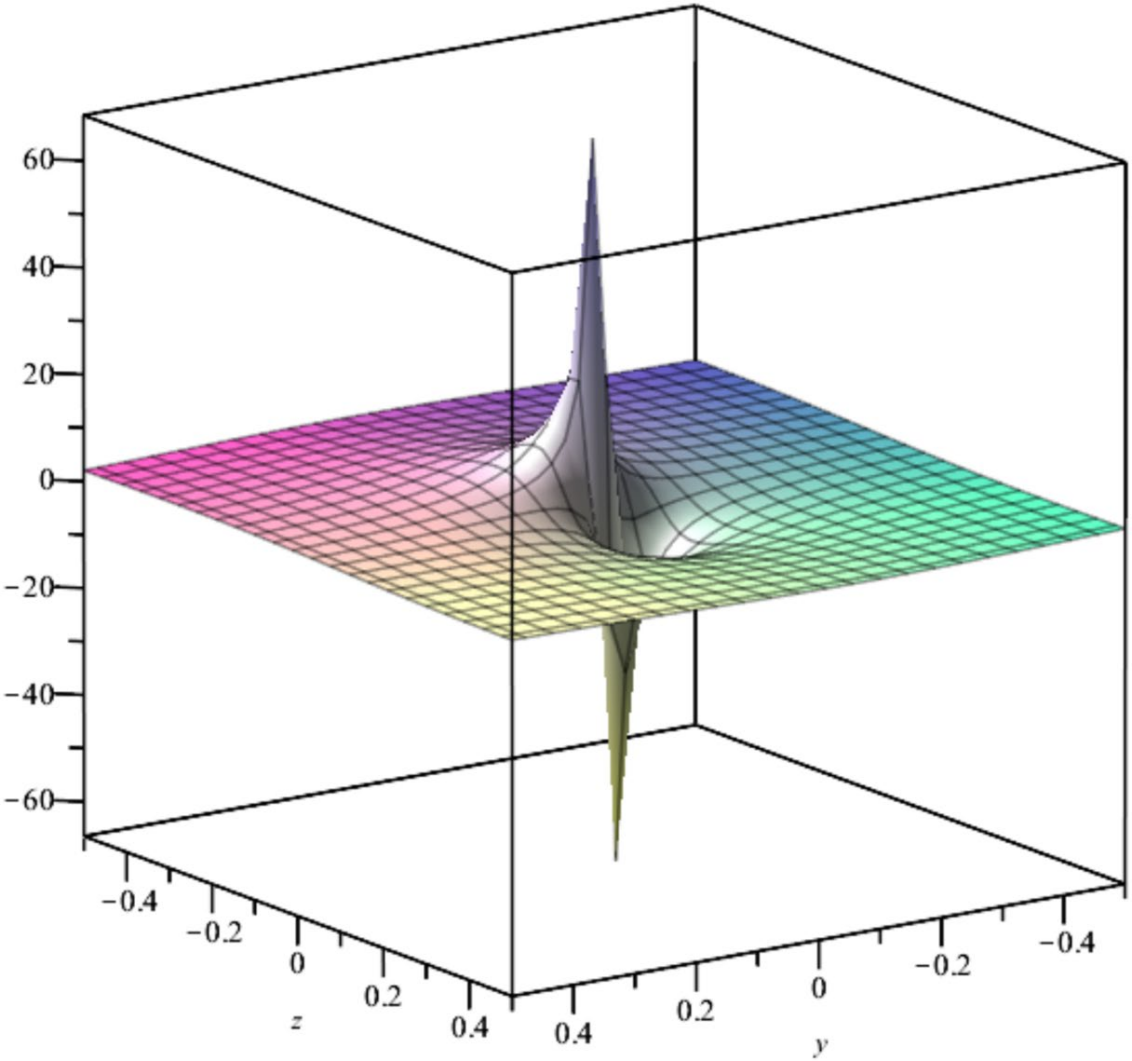
Illustration of ${u}_{8}$ at ${C}_{0}=1,{C}_{1}=1,{C}_{2}=-1, x=1,t=1$.

The new equation admits three infinitesimal Lie generators. The following subcases are derived and presented in Table 2. The solution, ${u}_{8}$​, is depicted in Fig. 8.

**Table 2:** Infinitesimal Vectors of Eq. (21) Using ${\text{X}}_{13}$.

Case 6.1	Vector	${q}^\frac{1}{3}\frac{\partial }{\partial r}+\frac{1}{3}{q}^{\frac{-2}{3}}\frac{\partial }{\partial w}.$
	Reduced Form	$36{o}^{2}\left({s}^{2}+1\right){v}_{ss}+81{o}^{4}{v}_{oo}+108s{o}^{3}{v}_{os}+243{o}^{3}{v}_{o}+144s{o}^{2}{v}_{s}+72{o}^{2}v-=0.$
	Solution	$v\left(s,o\right)=\frac{{{C}_{0}{s}^{2}+2C}_{2}s+2{C}_{1}}{2\left({s}^{2}+1\right)}+\frac{2-9{C}_{0}{o}^{2}}{18{o}^{2}}+{C}_{1}{o}^{\frac{-2}{3}}+{C}_{2}{o}^{\frac{-4}{3}}.$
	Final Solution of (1)	${u}_{8}=\frac{1}{{t}^\frac{7}{3}\left({y}^{2}+{z}^{2}\right)}\left({t}^\frac{7}{3}\left(-\frac{{y}^{2}}{3}+y\left({C}_{1}-\frac{{C}_{0}}{2}\right)-\frac{z\left(z-3{C}_{2}\right)}{3}\right)+\left({y}^{2}+{z}^{2}\right)\left({C}_{2}ty+\frac{1}{9}{t}^\frac{1}{3}{y}^{2}+\frac{1}{3}x{t}^\frac{4}{3}+{C}_{1}{t}^\frac{5}{3}\right)\right).$
Case 6.2	Vector	${q}^\frac{2}{3}\frac{\partial }{\partial p}-\left(\frac{p}{3{q}^\frac{1}{3}}\right)\frac{\partial }{\partial r}+\left(\frac{p}{9{q}^\frac{4}{3}}\right)\frac{\partial }{\partial w}.$
	Reduced Form 1	$36{s}^{2}{v}_{oooo}+9{s}^{2}\left({o}^{2}+4v\right){v}_{oo}+\left(54o{s}^{3}+36{s}^{2}\right){v}_{os}+81{s}^{4}{v}_{ss}+36{s}^{2}{v}_{o}^{2}+\left(63o{s}^{2}+12s\right){v}_{o}+243{s}^{3}{v}_{s}+72{s}^{2}v+4=0.$
	New level vector	${s}^\frac{1}{3}\frac{\partial }{\partial o}+\frac{1}{3}{s}^{\frac{-2}{3}}\frac{\partial }{\partial v}.$
	Reduced Form 2	$9{\alpha }^{2}{\phi }_{\alpha \alpha }+27\alpha {\phi }_{\alpha }+8\phi =0$
	Solution	$\phi \left(\alpha \right)={C}_{1}{\alpha }^{\frac{-2}{3}}+{C}_{2}{\alpha }^{\frac{-4}{3}}.$
	Final Solution of (1)	${u}_{9}=9{C}_{1}{t}^{\frac{-2}{3}}+9{C}_{2}y{t}^{\frac{-4}{3}}-\frac{1}{3}+\frac{x}{3t}+\frac{1}{9}{t}^{-2}{z}^{2}.$
Case 6.3	Vector	$\left({p}^{2}+1\right)\frac{\partial }{\partial p}+\left(\frac{3pq}{2}\right)\frac{\partial }{\partial q}+\left(\frac{pr}{2}\right)\frac{\partial }{\partial r}-\left(pw\right)\frac{\partial }{\partial w}.$
	Reduced Form 1	$4{v}_{oooo}+\left({o}^{2}+4v\right){v}_{oo}+6os{v}_{os}+9{s}^{2}{v}_{ss}+5o{v}_{o}+21s{v}_{s}+4{v}_{o}^{2}+4v=0.$
	New level vector	${s}^\frac{1}{3}\frac{\partial }{\partial o}+\frac{1}{3}{s}^{\frac{-2}{3}}\frac{\partial }{\partial v}.$
	Reduced Form 2	$81{\alpha }^{4}{\phi }_{\alpha \alpha }+189{\alpha }^{3}{\phi }_{\alpha }+36{\alpha }^{2}\phi -8=0.$
	Solution	$\phi \left(\alpha \right)={C}_{1}{\alpha }^{\frac{-2}{3}}\text{ln}\left(\alpha \right)+{C}_{2}{\alpha }^{\frac{-2}{3}}+\frac{1}{18{\alpha }^{2}}.$
	Final Solution of (1)	${u}_{10}=\frac{1}{36{t}^\frac{8}{3}}\left(-12{t}^\frac{8}{3}+12x{t}^\frac{5}{3}+36{C}_{1}{t}^{2}\text{ln}\left(t\right)-27{C}_{1}{t}^{2}\text{ln}\left({y}^{2}+{z}^{2}\right)+2{t}^\frac{2}{3}{y}^{2}+2{t}^\frac{2}{3}{z}^{2}+36{C}_{2}{t}^{2}\right).$

### ***Case 7: using***${{\varvec{X}}}_{16}$

This vector results in three distinct solutions that are given in the following forms:

26 $${u}_{11}={C}_{1}{t}^{\frac{-2}{3}}\mathit{ln}\left(t\right)-\frac{3}{4}{C}_{1}{t}^{\frac{-2}{3}}\mathit{ln}\left({y}^{2}+{z}^{2}+2z+1\right)+\frac{x}{3t}+\frac{{y}^{2}+{z}^{2}}{18{t}^{2}}+{C}_{2}{t}^{\frac{-2}{3}}+\frac{2z+1}{18{t}^{2}}-1.$$

27 $${u}_{12}=\frac{\left(-{y}^{2}+y\left({C}_{1}-\frac{{C}_{0}}{2}\right)-\left(z+1\right)\left(z+1-{C}_{2}\right)\right)}{\left({y}^{2}+{\left(z+1\right)}^{2}\right)}+{t}^{\frac{-7}{3}}\left({C}_{2}ty+\frac{1}{9}{t}^\frac{1}{3}{y}^{2}+\frac{x}{3}{t}^\frac{4}{3}+{C}_{1}{t}^\frac{5}{3}\right).$$

28 $${u}_{13}=\frac{x}{3t}+{C}_{2}{t}^{\frac{-2}{3}}+\frac{{y}^{2}}{9{t}^{2}}+{C}_{1}{t}^{\frac{-4}{3}}\left(z+1\right)-1.$$

### ***Case 8: using***${{\varvec{X}}}_{18}$

Another three exact solutions can be obtained as follows:

29 $$\begin{aligned} u_{{14}} = & \,\frac{4}{{t^{{\frac{7}{3}}} \left( {4t^{2} - 4tz + y^{2} + z^{2} } \right)}}\left( {t^{{\frac{7}{3}}} \left( { - \frac{{2z^{2} }}{3} + z\left( {\frac{{C_{2} }}{4} - \frac{x}{3}} \right) - \frac{{2y}}{9}\left( {y - \frac{9}{8}C_{1} - \frac{{C_{0} }}{{64}}} \right)} \right)} \right. \\ & \quad + t^{{\frac{4}{3}}} \left( {\frac{{xy^{2} + xz^{2} }}{{12}} - \frac{{zy^{2} }}{{36}} + \frac{{z^{3} }}{{12}}} \right) + t^{{\frac{{10}}{3}}} \left( {\frac{x}{3} + \frac{{5z}}{3} - \frac{{C_{2} }}{2}} \right) + \frac{{C_{1} }}{4}t^{{\frac{5}{3}}} \left( {y^{2} + z^{2} } \right) \\ & \quad + C_{1} t^{{\frac{{11}}{3}}} - \frac{4}{3}t^{{\frac{{13}}{3}}} + C_{1} zt^{{\frac{8}{3}}} + t^{{\frac{1}{3}}} \left( {\frac{{y^{2} z^{2} + y^{4} }}{{36}}} \right) + C_{2} yt^{3} - C_{2} yt^{2} z + \frac{{C_{2} yt}}{4}\left( {y^{3} + yz^{2} } \right) \\ \end{aligned}$$

30 $${u}_{15}={C}_{1}{t}^{\frac{-2}{3}}\text{ln}\left(4{t}^{2}-4tz+{y}^{2}+{z}^{2}\right)-\frac{{4C}_{1}}{3}{t}^{\frac{-2}{3}}\text{ln}\left(t\right)+{C}_{2}{t}^{\frac{-2}{3}}+\frac{x}{3t}+\frac{z}{9t}+\frac{{y}^{2}+{z}^{2}}{18{t}^{2}}-\frac{10}{9}.$$

31 $${u}_{16}={C}_{2}{t}^{\frac{-2}{3}}+{C}_{1}y{t}^{\frac{-4}{3}}+\frac{x}{3t}-\frac{z}{9t}+\frac{{z}^{2}}{9{t}^{2}}-\frac{8}{9}.$$

### Reduction using dual linear combinations of the vectors

Linear combinations of two vectors are used to create more solutions. The optimal combinations are ${X}_{6}+{X}_{10}, {X}_{7}+{X}_{10}, {X}_{10}+{X}_{12}, {X}_{7}+{X}_{16}, {X}_{9}+{X}_{16}, {X}_{13}+{X}_{16}$ and ${X}_{13}+{X}_{18}$. The reductions and solutions are discussed below.

### ***Case 1: using***${{\varvec{X}}}_{6}+{{\varvec{X}}}_{10}$

The combined vectors result in the following invariant variables:

32 $$p=t, q=-\frac{z+{z}^{2}+{y}^{2}}{2}, r=x+\frac{t}{2}\text{arctan}\left(\frac{2y}{-2z-1}\right), w\left(p,q,r\right)=u\left(x,y,z,t\right)+\frac{1}{2}\text{arctan}\left(\frac{2y}{-2z-1}\right).$$

Equation (1) is now transformed to:

33 $$\left(32q-4\right){w}_{rrrr}+\left(\left(32q-4\right)w-4{p}^{2}+32q-4\right){w}_{rr}-\left(64q-8\right)\left(\left(q-\frac{1}{8}\right)\left({w}_{qq}\right)-\frac{1}{2}{w}_{r}^{2}+{w}_{q}-\frac{1}{2}{w}_{pr}\right)=0.$$

Now, Eq. (33) is transformed to:

34 $$-8o{v}_{oo}+{v}_{oo}-8{v}_{o}=0,$$

Where, $$o=q, s=p, v\left(s,o\right)=w\left(p,q,r\right)-\frac{r}{p}$$.

Finally, Eq. (34) has the following exact solution:

35 $$v\left(s,o\right)={F}_{1}\left(s\right)+\text{ln}\left(o-\frac{1}{8}\right){F}_{2}(o).$$

The final exact solution of Eq. (1) can be formulated as:

36 $${u}_{17}=\frac{x}{t}+{F}_{1}\left(t\right)-3\text{ln}\left(2\right){F}_{2}\left(t\right)+{F}_{2}\left(t\right)\text{ln}(-4{y}^{2}-4{z}^{2}-4z-1).$$

Where, ${F}_{1}\left(t\right)and {F}_{2}(t)$ are arbitrary functions in their argument.

**Fig. 9:**
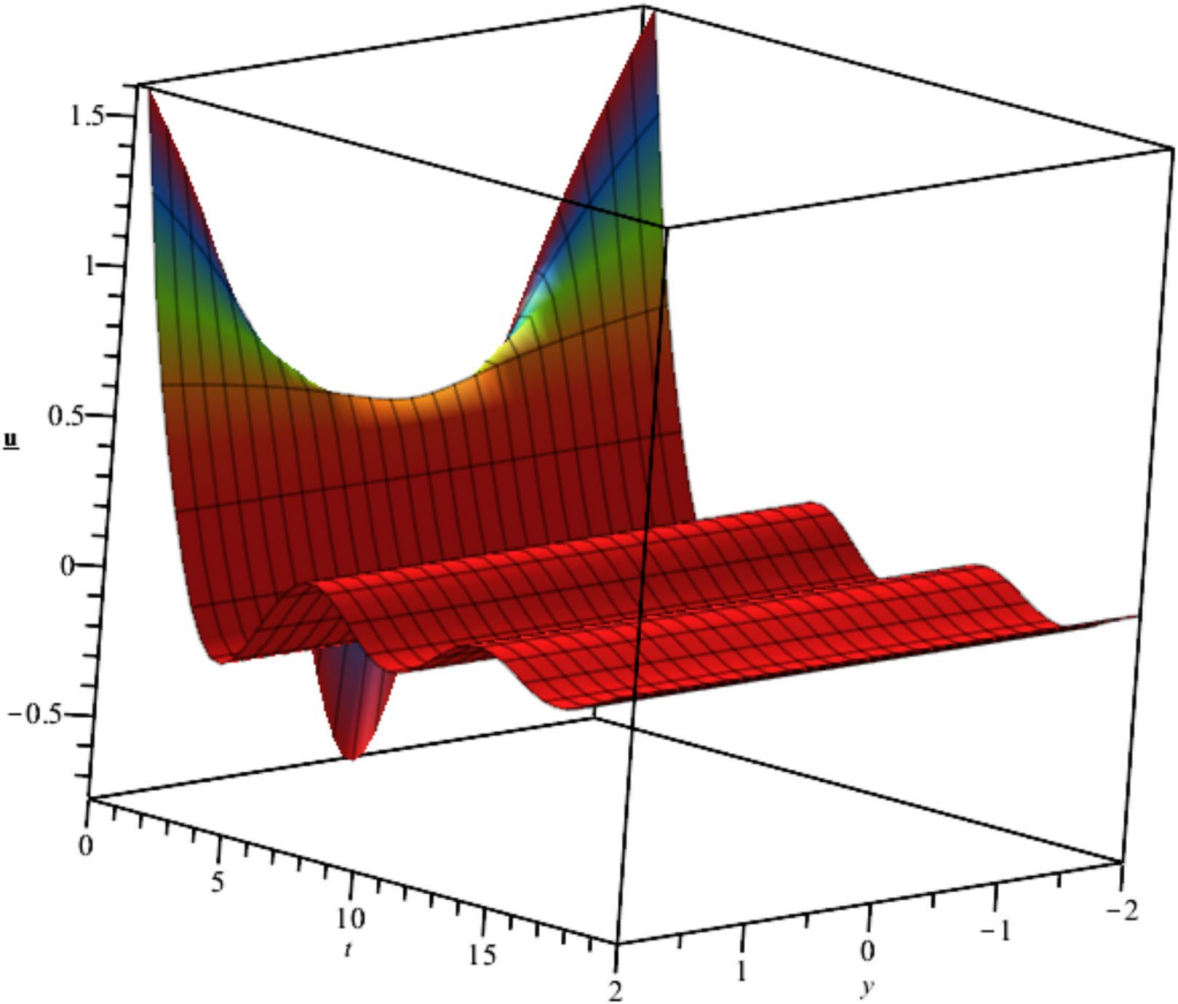
Illustration of ${u}_{17}$ at $z=0,x=0, {F}_{1}\left(t\right)=\frac{\text{sin}\left(t\right)}{t}, {F}_{2}\left(t\right)={e}^{-{t}^{2}}$.

The solution described by Eq. (36) is depicted in Fig. 9.

### ***Case 2: using***${{\varvec{X}}}_{7}+{{\varvec{X}}}_{10}$

The following solution is obtained.

37 $${u}_{18}=\left(\left(\frac{3}{4}{C}_{1}{t}^{\frac{-2}{3}}+\frac{1}{36{t}^{2}}\right)\left(1+2z-2y+2{z}^{2}+2y{y}^{2}\right)+\frac{1}{3t}\left(1+x\right)+\frac{3}{4}{C}_{1}\text{ln}\left(2\right){t}^{\frac{-2}{3}}+{C}_{1}{t}^{\frac{-2}{3}}\text{ln}\left(t\right)+36{C}_{2}{t}^{2}-1\right).$$

### ***Case 3: using***${{\varvec{X}}}_{10}+{{\varvec{X}}}_{12}$

The following solutions are obtained.

38 $${u}_{19}=\frac{{t}^{2}}{4}-\frac{z}{2}+{C}_{1}\text{ln}\left(4{y}^{2}+{\left({t}^{2}-2z\right)}^{2}\right)+{C}_{2}.$$

39 $${u}_{20}={C}_{1}-2{C}_{2}\text{ln}\left(2\right)-\frac{t}{4}+{C}_{2}\text{ln}\left({t}^{2}+t\left(2+4z\right)+4{y}^{2}+4{z}^{2}+4z+1\right).$$

**Fig. 10:**
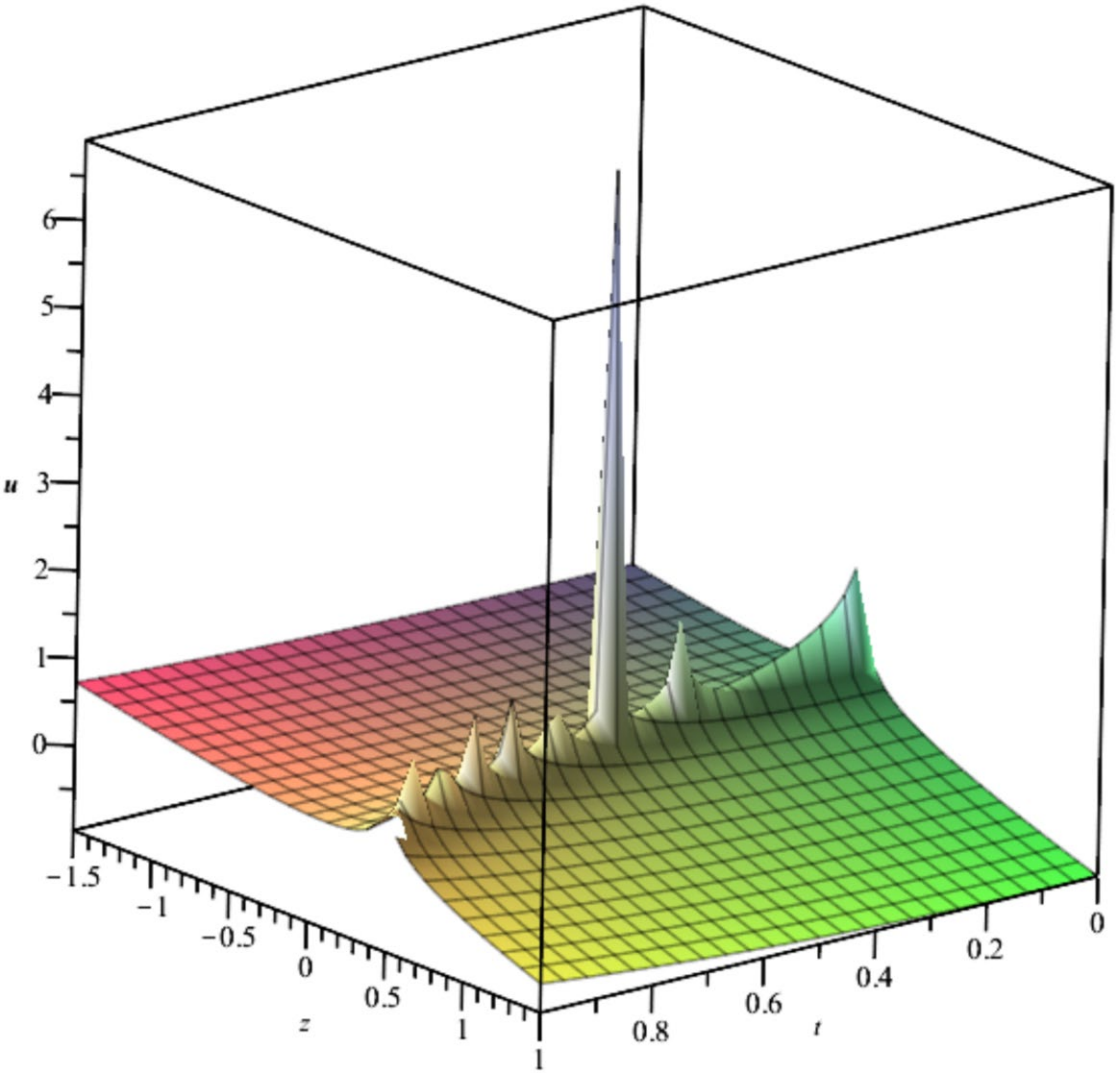
Illustration of ${u}_{19}$ at $y=0,x=0, {C}_{1}=0,{C}_{2}=-1$.

The solution, ${u}_{19}$, is illustrated in Fig. 10.

### ***Case 4: using***${{\varvec{X}}}_{7}+{{\varvec{X}}}_{16}$

The following solution has been created.

40 $$\begin{aligned} u_{{21}} = & \,\frac{{864}}{{\left( {3t + 2} \right)^{{\frac{7}{3}}} \left( {288t^{2} + t\left( {288 - 288z} \right) + 72y^{2} + 72\left( {z^{2} - 1} \right)} \right)}}\left( { - 4t^{4} + t^{3} \left( {x + 5z - \frac{{26}}{3} - \frac{3}{2}C_{2} } \right)} \right. \\ & \quad + t^{2} \left( { - \frac{2}{3}y^{2} + y\left( {\frac{{C_{0} }}{{96}} + \frac{3}{4}C_{1} } \right) + x\left( {\frac{5}{3} - z} \right) + C_{2} \left( {\frac{3}{4}z - \frac{{11}}{4}} \right) - 2z^{2} - 7 + \frac{{25}}{3}z} \right) \\ & \quad + t\left( {y^{2} \left( {\frac{x}{4} - \frac{z}{{12}} - \frac{5}{6}} \right) + y\left( {C_{1} + \frac{{C_{0} }}{{72}}} \right) + x\left( {\frac{{11}}{{12}} + \frac{{z^{2} }}{4} - \frac{{7z}}{6}} \right) + C_{2} \left( {z - \frac{5}{3}} \right) + 0.25\left( {z - 1} \right)\left( {z^{2} - \frac{{25}}{3}z + 10} \right)} \right) \\ & \quad + \frac{{y^{4} }}{{12}} + y^{2} \left( {\frac{{z^{2} }}{{12}} + \frac{x}{6} - \frac{1}{4}} \right) + y\left( {\frac{{C_{0} }}{{216}} + \frac{{C_{1} }}{3}} \right) + \frac{1}{6}\left( {z - 1} \right)\left( {2 + 2C_{2} - 3z + z^{2} + x\left( {z - 1} \right)} \right) \\ & \quad \left. { + \left( {3t + 2} \right)^{{\frac{1}{3}}} + \left( {t + \frac{2}{3}} \right)\left( {3^{{\frac{2}{3}}} C_{2} \left( {3t + 2} \right)^{{\frac{2}{3}}} + 3^{{\frac{4}{3}}} C_{1} y} \right)\left( {t^{2} + t\left( {1 - z} \right) + \frac{{y^{2} + \left( {z - 1} \right)^{2} }}{4}} \right)} \right). \\ \end{aligned}$$

**Fig. 11:**
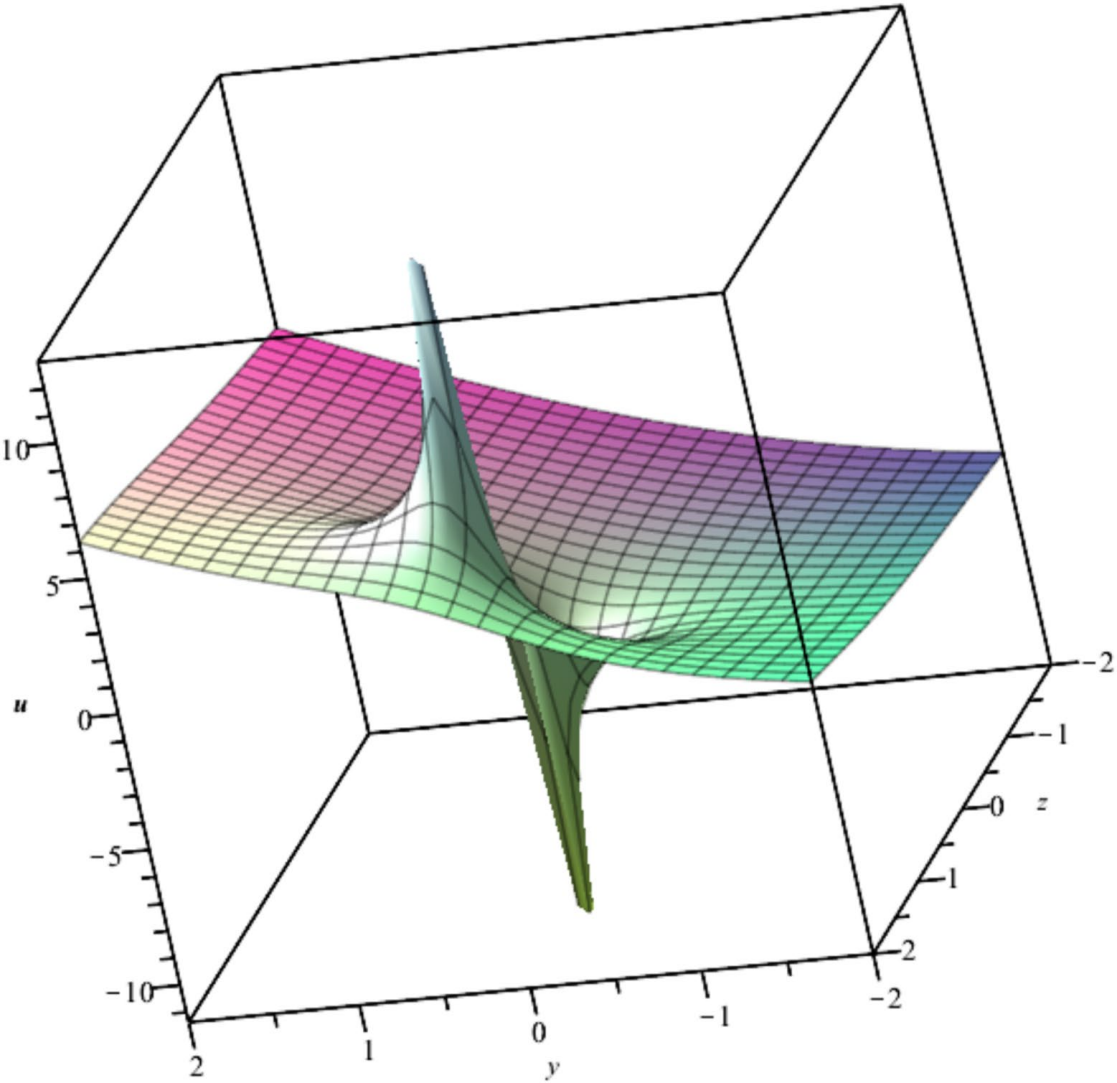
Illustration of ${u}_{20}$ at $t=0,x=0, {C}_{0}=0,{C}_{1}={C}_{2}=1.$

This solution is depicted in Fig. 11 at ${C}_{0}=0, {C}_{1}={C}_{2}=1,x=0,t=0$.

### ***Case 5: using***${{\varvec{X}}}_{13}+{{\varvec{X}}}_{16}$

Employing the same reduction procedures, the following solution is created.

41 $$\begin{aligned} u_{{21}} = & \,\frac{1}{{t^{{\frac{7}{3}}} \left( {y^{2} + \left( {z + \frac{1}{2}} \right)^{2} } \right)}}\left( {t^{{\frac{7}{3}}} \left( { - \frac{2}{3}y^{2} + y\left( {C_{1} - \frac{{C_{0} }}{2}} \right) - \frac{2}{3}\left( {z + \frac{1}{2}} \right)\left( {z - \frac{3}{2}C_{2} + \frac{1}{2}} \right)} \right)} \right. \\ & \quad \left. { + \left( {y^{2} + \left( {z + \frac{1}{2}} \right)^{2} } \right)\left( {C_{1} ty + \frac{1}{9}y^{2} t^{{\frac{1}{3}}} + \frac{x}{3}t^{{\frac{4}{3}}} + C_{2} t^{{\frac{5}{3}}} } \right)} \right). \\ \end{aligned}$$

**Fig. 12:**
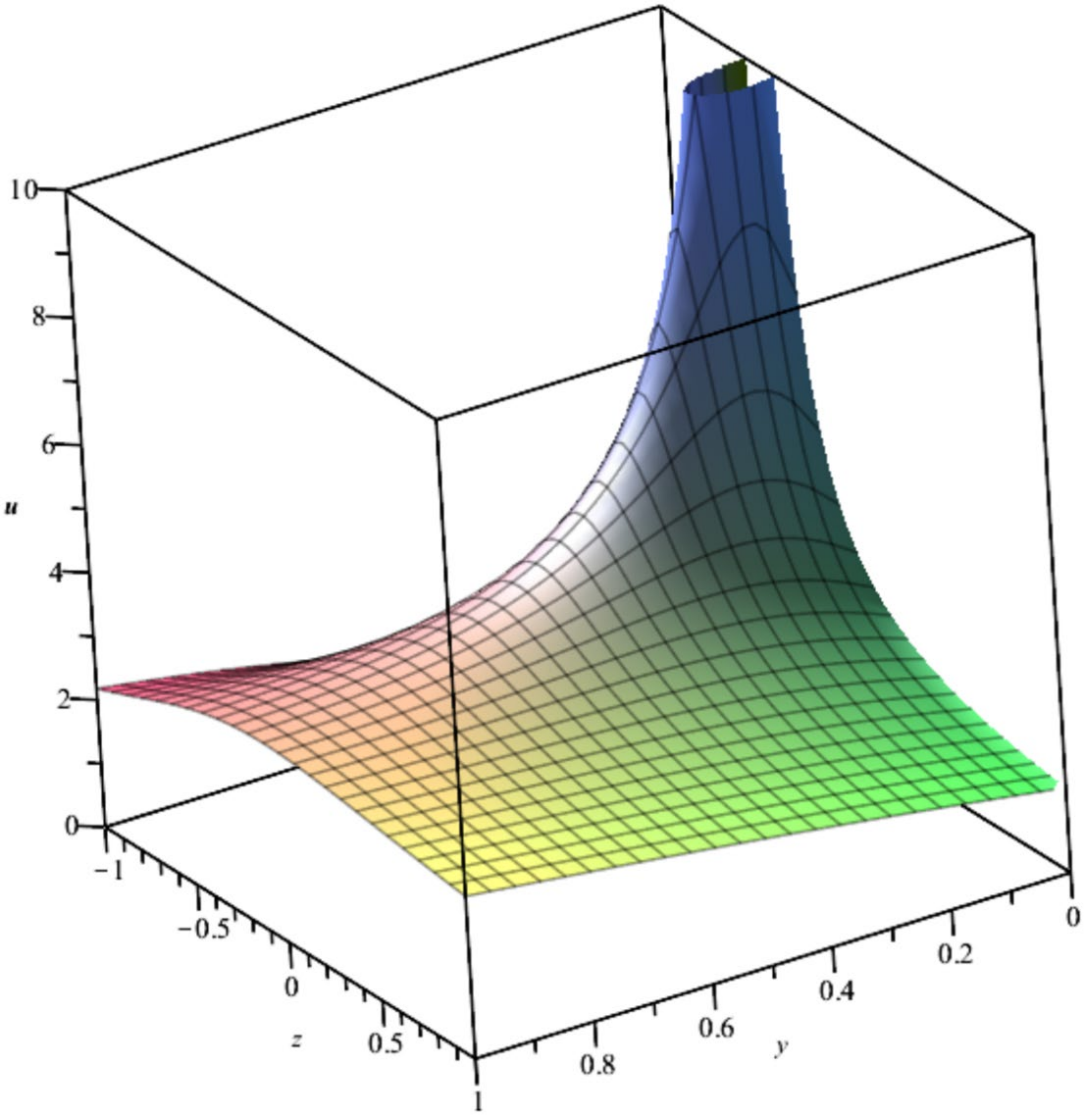
Illustration of ${u}_{21}$ at $t=1,x=1, {C}_{0}=0,{C}_{1}={C}_{2}=1.$

This solution is illustrated in Fig. 12 at $t=1,x=1, {C}_{0}=0,{C}_{1}={C}_{2}=1$.

### ***Case 6: using***${{\varvec{X}}}_{13}+{{\varvec{X}}}_{18}$

Another exact solution is formulated after the similarity transformation using the combined vectors, ${X}_{13}+{X}_{18}.$

42 $$\begin{aligned} u_{{22}} = & \,\frac{{27}}{{t^{{\frac{7}{3}}} \left( {72t^{2} - 144tz + 72y^{2} + 72z^{2} } \right)}}\left( {\left( { - \frac{{13}}{{12}}z^{2} + z\left( {C_{2} - \frac{{2x}}{3}} \right) - \frac{{23y}}{{36}}\left( {y - \frac{{36}}{{23}}C_{1} - \frac{{C_{0} }}{{46}}} \right)} \right)t^{{\frac{7}{3}}} } \right. \\ & \quad + t^{{\frac{4}{3}}} \left( {\frac{1}{3}xy^{2} + \frac{1}{3}xz^{2} - \frac{1}{{18}}y^{2} z + \frac{1}{6}z^{3} } \right) + t^{{\frac{{10}}{3}}} \left( {\frac{x}{3} + \frac{{5z}}{3} - C_{2} } \right) + t^{{\frac{5}{3}}} \left( {y^{2} + z^{2} } \right) \\ & \quad \left. { + C_{1} t^{{\frac{{11}}{3}}} - \frac{3}{4}t^{{\frac{{13}}{3}}} - 2C_{1} zt^{{\frac{8}{3}}} + t^{{\frac{1}{3}}} \left( {\frac{1}{9}y^{4} + \frac{1}{9}y^{2} z^{2} + C_{2} yt^{3} - 2C_{2} yt^{2} z + C_{2} ty^{3} + C_{2} tyz^{2} } \right)} \right). \\ \end{aligned}$$

**Fig. 13:**
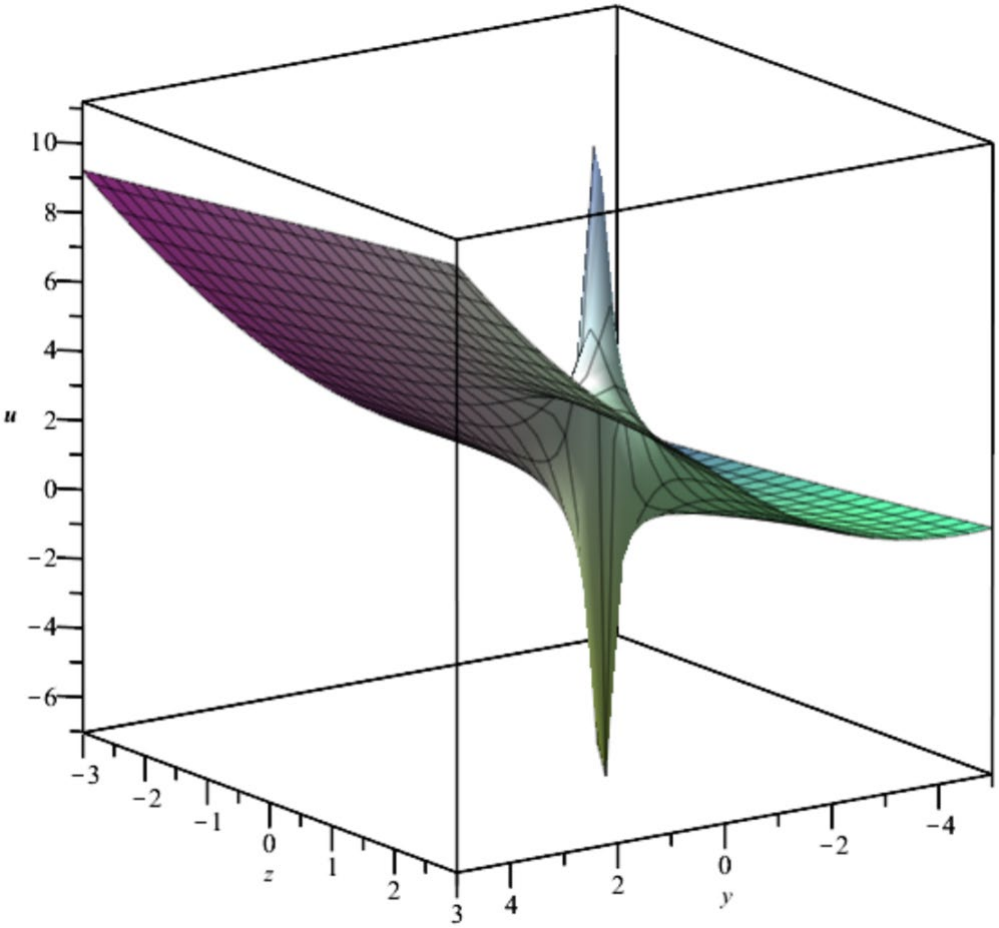
Illustration of ${u}_{22}$ at $t=1,x=5, {C}_{0}=0,{C}_{1}={C}_{2}=1$.

Now, this solution is depicted in Fig. 13.

### Reduction using triple linear combinations of vectors

Linear combinations of three vectors are used to create more solutions. The optimal combinations are ${X}_{6}+{X}_{7}+{X}_{10}, {X}_{6}+{X}_{7}+{X}_{12},{X}_{6}+{X}_{10}+{X}_{12},{X}_{7}+{X}_{10}+{X}_{12}, {X}_{9}+{X}_{13}+{X}_{18}, {X}_{13}+{X}_{16}+{X}_{18}$. The reductions and solutions are described hereafter.

### ***Case 1: using***${{\varvec{X}}}_{6}+{{\varvec{X}}}_{7}+{{\varvec{X}}}_{10}$

This linear combination leads to the following exact solution.

43 $${u}_{23}={F}_{2}\left(t\right)\text{ln}\left(-\frac{3}{2}{z}^{2}-\frac{3}{2}{y}^{2}-z+y-\frac{1}{3}\right)+{F}_{1}\left(t\right)-\frac{1}{3}\text{arctan}\left(\frac{3y-1}{-3z-1}\right).$$

**Fig. 14:**
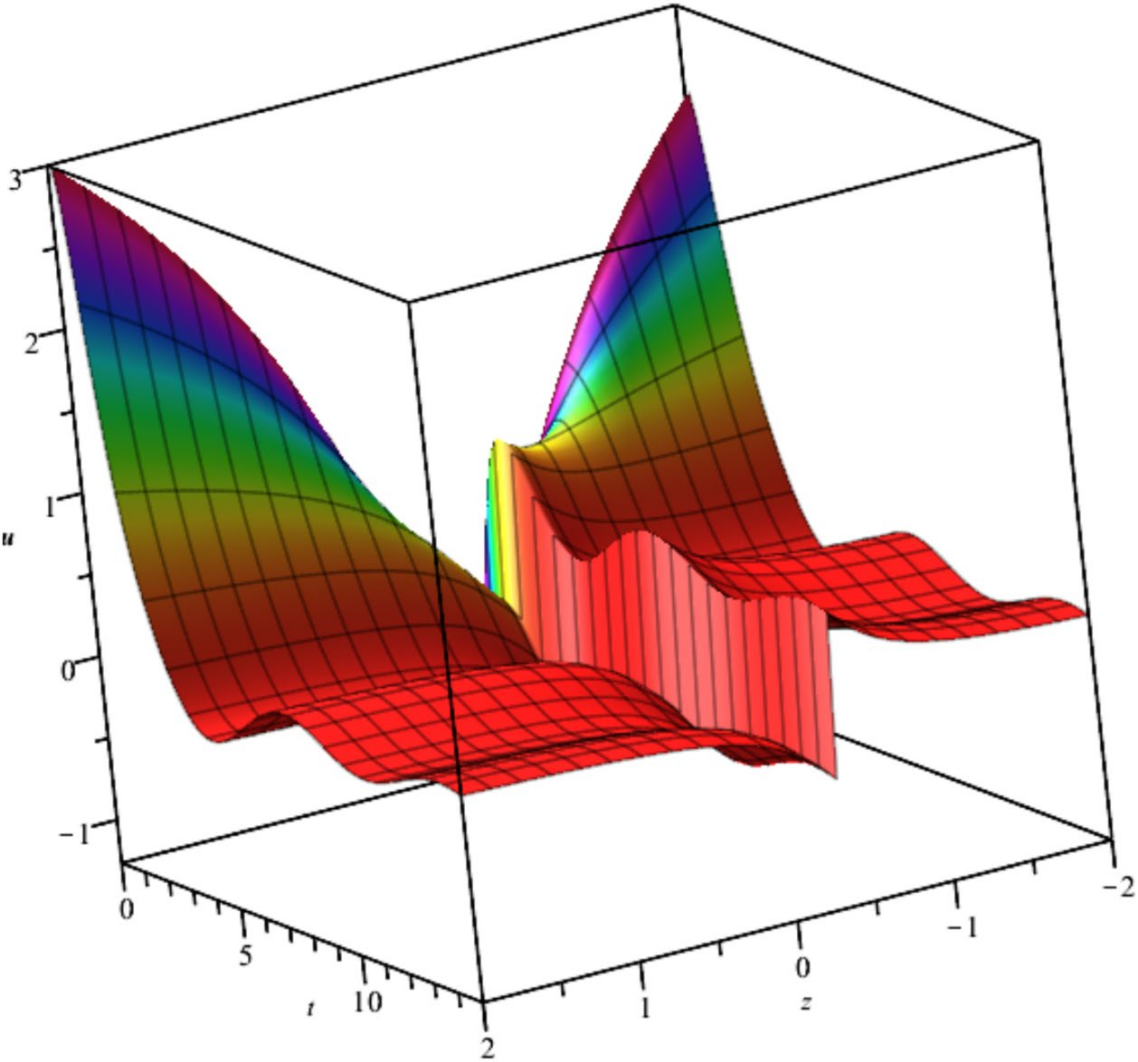
Illustration of ${u}_{23}$ at ${F}_{1}\left(t\right)={\text{sech}}^{2}(t), {F}_{2}\left(t\right)={e}^{-{t}^{2}}$ and $y=0$.

The solution is depicted in Fig. 14 considering ${F}_{1}\left(t\right)={\text{sech}}^{2}(t), {F}_{2}\left(t\right)={e}^{-{t}^{2}}$ at $y=0$.

### ***Case 2: using***${{\varvec{X}}}_{6}+{{\varvec{X}}}_{7}+{{\varvec{X}}}_{12}$

This linear combination leads to the following exact solution.

44 $${u}_{24}={F}_{2}\left(t\right)\text{ln}\left(-\frac{1}{6}{t}^{2}-\frac{3}{2}{z}^{2}-zt-\frac{3}{2}{y}^{2}+y-\frac{1}{6}\right)+{F}_{1}\left(t\right)-\frac{1}{3}\text{arctan}\left(\frac{3y-1}{-3z-t}\right).$$

**Fig. 15:**
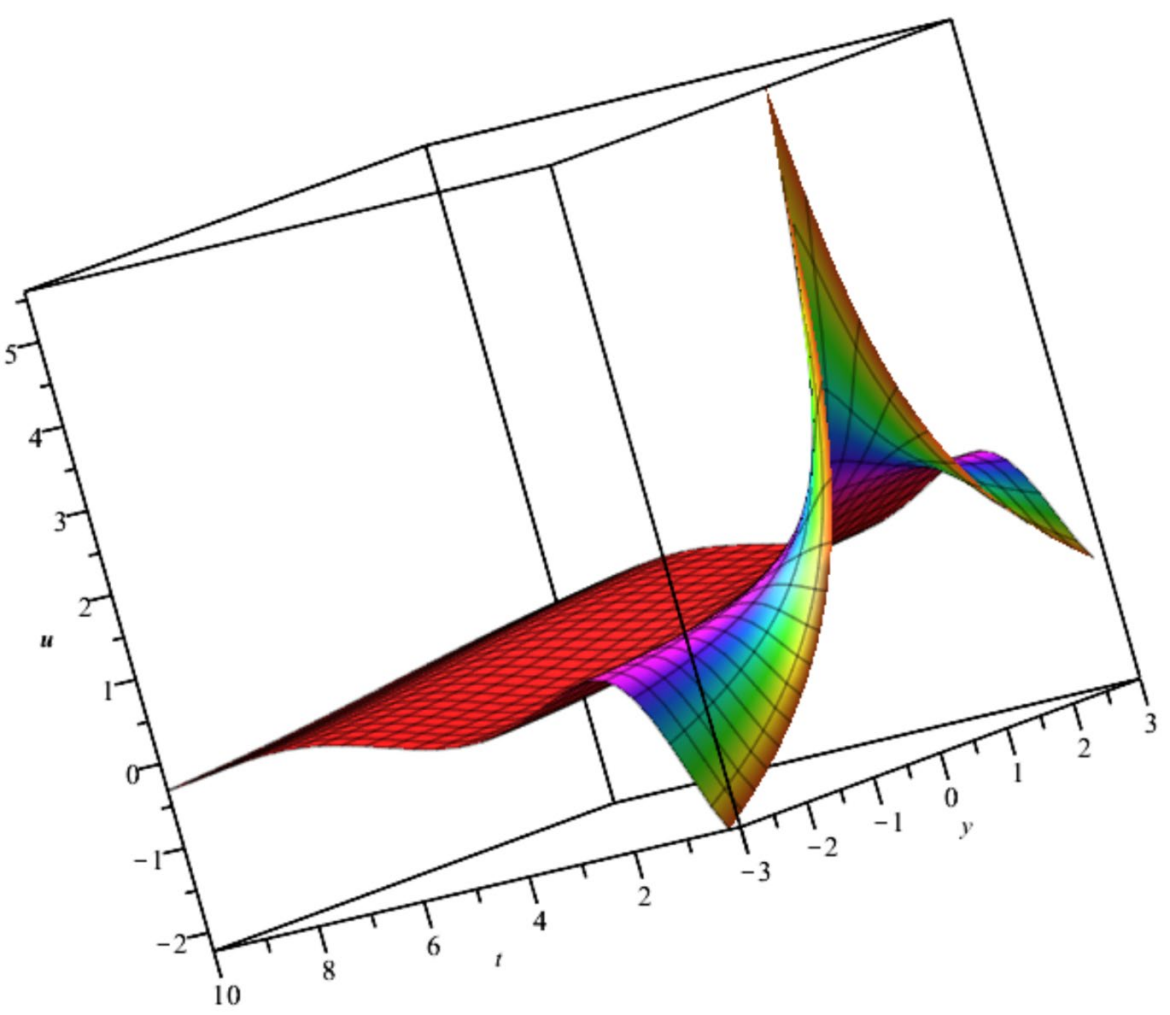
Illustration of ${u}_{24}$ at ${F}_{1}\left(t\right)={\text{sech}}^{2}(t), {F}_{2}\left(t\right)=\frac{\text{sin}\left(t\right)}{t}$ and $z=0.$

The solution is depicted in Fig. 15 cosideringing ${F}_{1}\left(t\right)={\text{sech}}^{2}(t), {F}_{2}\left(t\right)=\frac{\text{sin}\left(t\right)}{t}$ at $z=0$.

### ***Case 3: using***${{\varvec{X}}}_{6}+{{\varvec{X}}}_{10}+{{\varvec{X}}}_{12}$

This linear combination leads to the following exact solution.

45 $${u}_{25}={F}_{2}\left(t\right)\text{ln}\left(-\frac{1}{18}{t}^{2}-\frac{1}{2}{z}^{2}-\frac{1}{3}zt-\frac{1}{2}{y}^{2}-\frac{1}{3}z-\frac{1}{9}t-\frac{1}{18}\right)+{F}_{1}\left(t\right)-\frac{1}{3}\text{arctan}\left(\frac{3y}{-3z-t-1}\right).$$

### ***Case 4: using***${{\varvec{X}}}_{7}+{{\varvec{X}}}_{10}+{{\varvec{X}}}_{12}$

Once again, this linear combination leads to the following exact solution.

46 $${u}_{26}={C}_{1}+{C}_{2}\text{ln}\left(-\frac{1}{6}{t}^{2}-\frac{1}{3}t-\frac{3}{2}{z}^{2}-zt-\frac{3}{2}{y}^{2}-z+y-\frac{1}{3}\right).$$

### ***Case 5: using***${{\varvec{X}}}_{9}+{{\varvec{X}}}_{8}+{{\varvec{X}}}_{13}$

The following solution is obtained.

47 $$\begin{aligned} u_{{27}} = & \,\frac{1}{{6\left( {3t + 1} \right)^{2} }}\left( {6\left( {3^{{\frac{2}{3}}} } \right)C_{1} \left( {3t + 1} \right)^{{\frac{4}{3}}} {\text{ln}}\left( {4t^{2} + t\left( {4 - 4z} \right) + y^{2} + z^{2} - 2z + 1} \right)} \right. \\ & \quad \left. { + 8\left( {3^{{\frac{2}{3}}} } \right)\left( {3t + 1} \right)^{{\frac{4}{3}}} \left( {C_{1} {\text{ln}}\left( 3 \right) - C_{1} {\text{ln}}\left( {3t + 1} \right) + \frac{3}{4}C_{2} } \right) - 42t^{2} + t\left( {18x + 6z - 42} \right) + 3y^{2} + 3z^{2} + 6x - 9} \right). \\ \end{aligned}$$

48 $${u}_{28}=\frac{3}{{\left(3t+1\right)}^\frac{7}{3}}\left({\left(3t+1\right)}^\frac{1}{3}\left(-\frac{5}{3}{t}^{2}+t\left(x-\frac{z}{3}-\frac{5}{3}\right)+\frac{{z}^{2}}{3}+\frac{x-z+1}{3}\right)+\left(t+\frac{1}{3}\right)\left({C}_{2}{\left(3\right)}^\frac{2}{3}{\left(3t+1\right)}^\frac{2}{3}+{3}^\frac{4}{3}{C}_{1}y\right)\right).$$

### ***Case 6:***${{\varvec{X}}}_{13}+{{\varvec{X}}}_{16}+{{\varvec{X}}}_{18}$

This last triple combination leads to the following result.

49 $$\begin{aligned} _{{29}} = & \,\frac{1}{{162t^{2} }}\left( {162C_{1} t^{{\frac{4}{3}}} {\text{ln}}\left( {4t^{2} + t\left( { - 4 - 12z} \right) + 9y^{2} + 9z^{2} + 6z + 1} \right)} \right. \\ & \quad \left. { + t^{{\frac{4}{3}}} \left( { - 324{\text{ln}}\left( 3 \right)C_{1} - 216C_{1} {\text{ln}}\left( t \right) + 162C_{2} } \right) - 128t^{2} + t\left( {54x + 6z + 8} \right) + 9y^{2} + 9z^{2} + 6z + 1} \right). \\ \end{aligned}$$

### Reduction using quadruple linear combination vectors

A linear combination of four vectors is used to create more solutions. The optimal set of combinations consists of only one combination, ${X}_{12}+{X}_{13}+{X}_{16}+{X}_{18}$. The solution resulting from using this combination is formulated as:

50 $$\begin{aligned} u_{{30}} = & \,F_{2} \left( t \right){\text{ln}}\left( { - t^{2} - t\left( {2 + 8z} \right) - 16y^{2} - 16z^{2} + 8y - 8z - 2} \right) \\ & \quad - 3{\text{ln}}\left( 2 \right)F_{2} \left( t \right) + F_{1} \left( t \right) + \frac{1}{{32t}}arctan\left( {\frac{{4y - 1}}{{t + 4z + 1}}} \right) + \frac{x}{t} - \frac{z}{{8t}} - \frac{1}{{32t}} - \frac{1}{{32}}. \\ \end{aligned}$$

**Fig. 16:**
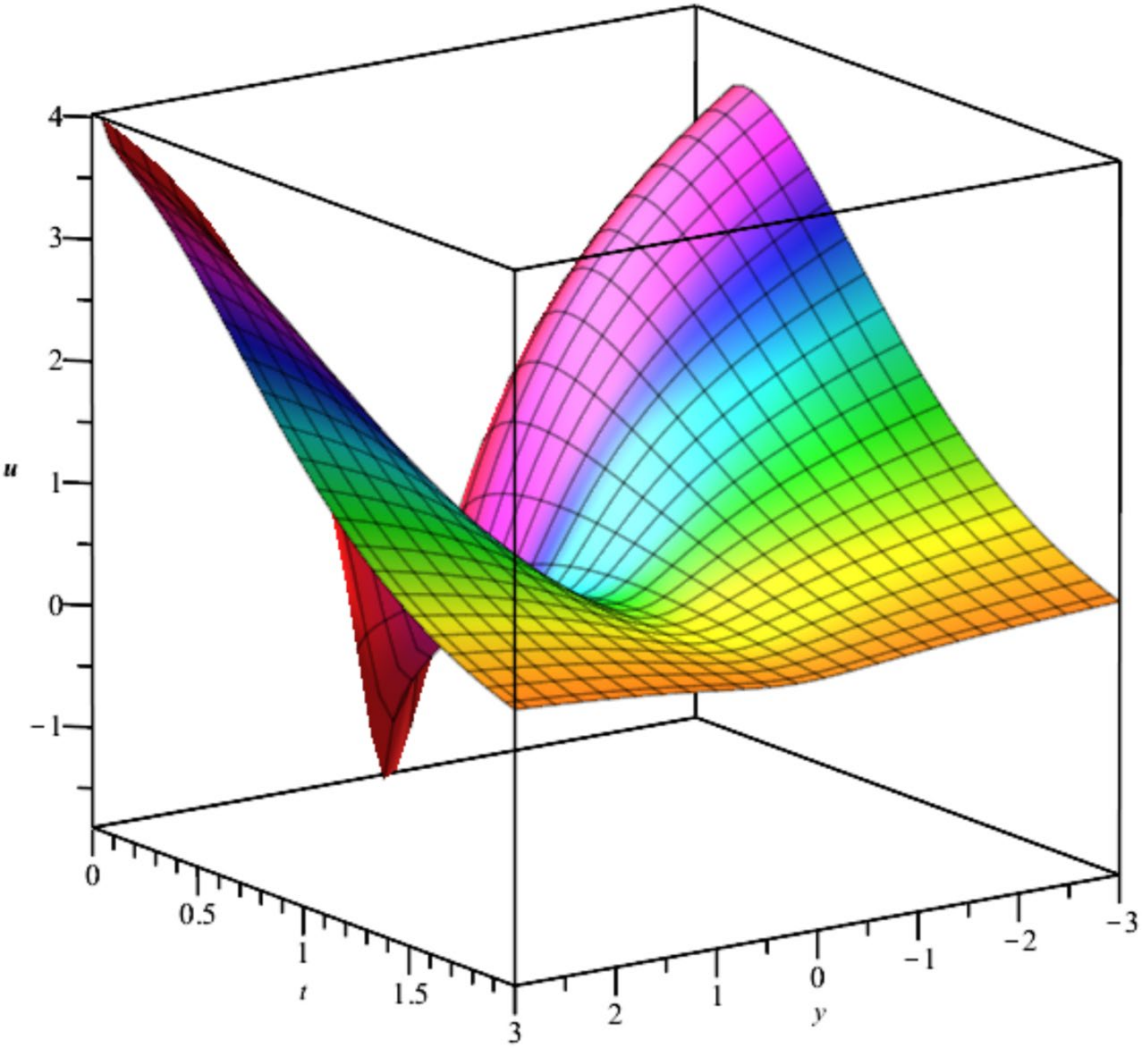
Illustration of ${u}_{30}$ at ${F}_{1}\left(t\right)={\text{sech}}^{2}(t), {F}_{2}\left(t\right)=\text{sech}(t)$ and $x=0,z=0$.

This solution is depicted hereafter in Fig. 16.

## Discussion of the results

Bubble creation in liquids is a widespread phenomenon with extensive influence on natural and industrial systems. It facilitates mixing, heat, and mass transport and is therefore essential in processes like chemical reactions, wastewater treatment, and fermentation. In nature, bubbling occurs as submarine volcanism and as part of aquatic life activity. Its dynamics also enable sophisticated applications like ultrasound imaging and drug targeting. Phenomena of gas bubbling need to be mastered to enhance efficiency, safety, and innovation in a multitude of scientific and engineering processes. The optimal system emphasizes obtaing nonrepeated exact solutions^67,68^. The obtained illustrations, Figs. 1, 2, 3, 4, 5, 6, 7, 8, 9, 10, 11, 12, 13, 14, 15 and 16, introduce many scenarios of bubble formation in fluids. Figure 1 illustrates gas bubbling through a liquid, with increasing wave-like tendencies in velocity over time and space due to rising gas bubbles that agitate the surrounding liquid. These are typical situations in chemical reactors, bubble columns, and bioreactors, where gas–liquid interaction is a significant factor. Such knowledge can optimize mixing, mass transfer, and reaction rates in industrial processes. Figure 2 presents a pressure or velocity wave propagating in a fluid medium and peaking abruptly. In such cases, a gas bubble rising in a fluid generates oscillatory pressure fields through displacement and buoyant forces. Applications include chemical reactors, underwater acoustics, and medical diagnostics involving microbubbles. Figures 3, 5, 6 and 7 depict several high-frequency oscillating waves with clear peaks and troughs, which are characteristic of complex wave interactions. In gas bubbling in liquids, for instance, such graphs may model turbulence or stochastic waveforms resulting from large numbers of bubbles interacting in a confined area. Sonar and medical ultrasound devices are common applications. Figure 4 displays a single wave representing pressure or velocity distribution caused by the expansion and distortion of a single gas bubble in a viscous liquid. Such waveforms are used to model the initial burst or implosion of bubbles and play a critical role in heat and mass transfer. Fluidized beds, microfluidic devices, and safety analysis in pressurized gas–liquid systems are just a few relevant application areas. Figures 8, 11, and 13 show waves that are likely singularities or steep gradients in fluid behavior, possibly mimicking the pressure or velocity fields around a gas bubble in a fluid. Such dynamics are significant in chemical reactors, underwater acoustics, and biomedical ultrasound. Figure 9 illustrates wave decay with energy loss over time. Applications range from improving aeration in wastewater treatment to optimizing gas delivery in biomedical therapies. Figure 10 shows a localized spike, as observed in gas bubble collapse or microbubble cavitation within a fluid. The transient spike in amplitude suggests concentrated energy over a short timescale, relevant in high-pressure or ultrasonic systems. This is crucial in applications such as medical ultrasound therapy, inkjet printing, and fuel injection systems, where precise bubble dynamics determine performance. In Fig. 11, peaks and steep slopes indicate regions of high intensity—for example, where bubbles rise and create local disturbances. Such wave-like structures can be used to study gas–liquid interactions, mixing efficiency, and bubble plume dynamics. Applications include chemical reactors, wastewater treatment, and bubble column reactor design. Figure 14 reveals oscillations or perturbations at the gas–liquid interface during gas bubbling. The sharp vertical ridges and oscillating surface represent nonlinear wave dynamics or shock-like structures, typically resulting from abrupt gas injection into a viscous fluid. Applications include design optimization of gas sparging in bioreactors and efficiency improvements in fluidized beds and chemical mixing systems. Figures 15 and 16 present dynamic wave profiles, likely modeling fluid surface deformation over time due to gas bubbles rising through a liquid. The steep peaks and troughs indicate local, nonlinear disturbances such as bubble bursts or high-speed jets. Applications include the design of optimized bubble columns, aeration systems, and enhancement of gas–liquid reaction efficiency.

## Conclusion

The results obtained by the optimal Lie infinitesimals are divided into four main categories. The reductions and solutions are obtained using single, double, triple, and quadruple linear combinations of the vectors. The resulting closed-form solutions were verified symbolically using the MAPLE package to satisfy the NLWE. These solutions are numerous and diverse in form. Some are expressed as single and multiple solitons, while others appear as singular solitons, or as periodic, logarithmic, exponential, and polynomial waves. Moreover, some solutions are formulated in terms of arbitrary functions, which can be appropriately selected to represent various physical cases. These solutions are particularly important for understanding the behavior of bubbles in gaseous media under different conditions. The chaotic nature of the gaseous medium is the primary reason for the emergence of such varied solutions. The optimal system used is crucial for generating new exact solutions. Higher-order linear combinations lead to more complex solutions of the nonlinear wave equations. However, the number of optimal system members decreases as the order of the linear combination increases, that is, quadruple-vector combinations are fewer than triple, double, or single ones.

## Data Availability

All data generated or analysed during this study are included in this published article.
